# Four bioactive new steroids from the soft coral *Lobophytum pauciflorum* collected in South China Sea

**DOI:** 10.3762/bjoc.18.42

**Published:** 2022-04-08

**Authors:** Di Zhang, Zhe Wang, Xiao Han, Xiao-Lei Li, Zhong-Yu Lu, Bei-Bei Dou, Wen-Ze Zhang, Xu-Li Tang, Ping-Lin Li, Guo-Qiang Li

**Affiliations:** 1 Key Laboratory of Marine Drugs, Chinese Ministry of Education, School of Medicine and Pharmacy, Ocean University of China, Qingdao 266003, People's Republic of China; 2 Laboratory of Marine Drugs and Biological Products, Pilot National Laboratory for Marine Science and Technology, Qingdao 266235, People's Republic of China; 3 College of Chemistry and Chemical Engineering, Ocean University of China, Qingdao 266100, People's Republic of China

**Keywords:** anti-inflammatory, cytotoxicity, *Lobophytum pauciflorum*, soft coral, steroids, X-ray diffraction

## Abstract

Four new polyhydroxylated steroids lobophysterols E–H (**1**–**4**), together with three known compounds (**5**–**7**), were isolated from the soft coral *Lobophytum pauciflorum* collected at Xisha Island, China. The structures of the new compounds were elucidated by extensive spectroscopic analysis and comparison with NMR data of structurally related compounds reported in the literature. The absolute configuration of **1**–**3** was determined by X-ray diffraction. All the compounds have assessed the cytotoxicity against HL-60, K562, and Hela cells. Compound **1** showed weak cytotoxicity against K562 cells with an IC_50_ value of 19.03 μM. In addition, compound **1** also showed a moderate anti-inflammatory effect in zebrafish.

## Introduction

The unique and complicated marine environment makes soft corals a treasure-house of secondary metabolites with great variety and bioactivities. Previous chemical studies on soft corals *Lobophytum,* widely distributed in the world, resulted in the identification of lobane diterpene [1], cembranoids [2], and biscembranoids [3] with different bioactivities. Moreover, structurally specific steroids containing side chains with 23,24-dimethyl groups and (17)20*E* double bond, have been reported to be frequently isolated from soft corals of this genus, some of them exhibited anti-inflammatory [4], cytotoxic [5–6], and antibacterial activities [7].

**Figure 1:**
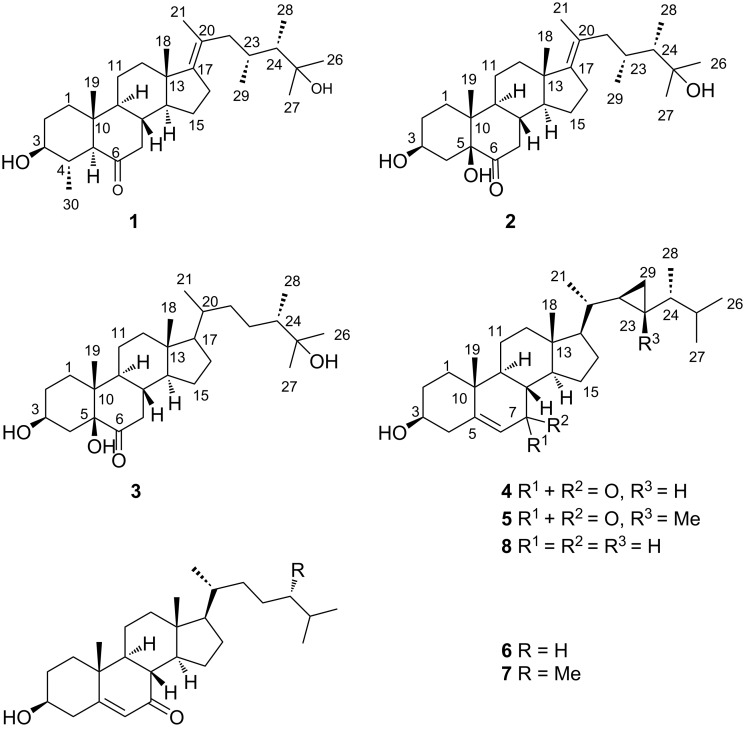
Structures of compounds **1**–**7**.

To search for bioactive natural products, we have investigated the chemical constituents of the soft coral *Lobophytum pauciflorum*, collected from Xisha Island in the South China Sea. In the present paper, we describe the isolation of four new polyhydroxylated steroids lobophysterols E–H (**1**–**4**), together with three known compounds (**5**–**7**) (Figure 1). The structure of the new compounds was established by extensive spectroscopic analysis and comparing with the spectroscopic data of the previously reported structurally-related compounds. Compounds **1** and **2** represented rare examples of steroids with both 23,24-dimethyl groups and 17(20)*E* double bond. In particular, compound **1** also has a tetracyclic skeleton with a methyl group at C-4. The absolute configuration of **1**–**3** was determined by X-ray analysis. Herein, we report the isolation, structure elucidation, and bioactivities of these compounds.

## Results and Discussion

Compound **1** was isolated as a white powder. Its molecular formula was established as C_30_H_50_O_3_ by HRESIMS from the molecular ion peak at *m*/*z* 481.3648 [M + Na]^+^. The ^1^H NMR data (Table 1) showed 5 methyl singlets (δ_H_ 0.76, CH_3_-19; δ_H_ 0.84, CH_3_-18; δ_H_ 1.19, CH_3_-26; δ_H_ 1.19, CH_3_-27; δ_H_ 1.67, CH_3_-21), 3 methyl doublets (δ_H_ 0.81, CH_3_-29; δ_H_ 0.84, CH_3_-28; δ_H_ 1.00, CH_3_-30), an oxymethine (δ_H_ 3.13, 1H, m) and a series of methylene multiplets located between δ_H_ 1.25 and δ_H_ 2.40. The ^13^C NMR and DEPT spectra exhibited the presence of 30 carbon signals, including a carbonyl group (δ_C_ 210.0), a tetrasubstituted double bond (δ_C_ 144.4 and 124.2), one oxygenated sp^3^ secondary carbon (δ_C_ 76.3) and one oxygenated sp^3^ quaternary carbon (δ_C_ 74.2). As two of the six degrees of unsaturation were occupied by the double bond and carbonyl group, the remaining four unsaturations of **1** corresponded to a tetracyclic skeleton.

**Table 1:** ^1^H and ^13^C NMR data of compounds **1**–**4** (δ in ppm and *J* in Hz).

No.	**1**		**2**		**3**		**4**	
	δ_C_^a^	δ_H_^b^	δ_C_^c^	δ_H_^d^	δ_C_^a^	δ_H_^b^	δ_C_^a^	δ_H_^b^

1	36.9, CH_2_	1.80, m; 1.79, m	25.9, CH_2_	1.79, m; 1.52, m	25.0, CH_2_	1.79, m; 1.49, m	36.5, CH_2_	1.22, m; 1.95, m
2	30.3, CH_2_	1.83, m	28.4, CH_2_	1.80, m; 1.66, m	28.0, CH_2_	1.75, m; 1.66, m	31.3, CH_2_	1.93, m; 1.61, m
3	76.3, CH	3.13, td (10.7, 4.6)	67.1, CH	4.06, m	65.7, CH	4.03, m	70.7, CH	3.68, m
4	34.3, CH	1.76, m	38.0, CH_2_	2.45, dd (14.4, 3.5); 1.62, m	37.3, CH_2_	2.26, m; 1.65, m	42.0, CH_2_	2.40, m; 2.50, m(br.)
5	64.0, CH	2.08, m	83.5, C		82.1, C		165.3, C	
6	210.0, C		213.5, C		212.9, C		126.2, CH	5.70, s
7	48.4, CH_2_	2.06, m; 2.32, m	43.0, CH_2_	2.41, m; 2.33, dd (14.1, 4.8)	41.6, CH_2_	2.40, dd (14.1, 3.6); 2.22, m	202.6, C	
8	39.1, CH	1.85, m	37.7, CH	1.83, m	37.5, CH	1.76, m	45.6, CH	2.23, m
9	54.7, CH	1.26, m	43.7, CH	1.94, m	43.0, CH	1.75, m	50.1, CH	1.53, m
10	43.1, C		45.2, C		44.2, C		38.5, C	
11	22.1, CH_2_	1.36, m; 1.67, m	23.1, CH_2_	1.49, m; 1.67, m	21.8, CH_2_	1.36, m; 1.54, m	21.4, CH_2_	1.57, m
12	37.7, CH_2_	2.32, m	38.8, CH_2_	2.39, m	39.6, CH_2_	2.05, m	38.8, CH_2_	1.14, m; 2.03, m
13	45.2, C		46.1, C		43.3, C		43.5, C	
14	56.8, CH	1.33, m	57.7, CH	1.50, m	57.0, CH	1.26, m	49.8, CH	1.32, m
15	24.4, CH_2_	1.56, m	25.2, CH_2_	1.64, m; 1.23, m	24.1, CH_2_	1.54, m; 1.08, m	26.8, CH_2_	2.42, m; 1.23, m
16	29.9, CH_2_	2.15, m; 2.33, m	30.8, CH_2_	2.22, m; 2.38, m	28.0, CH_2_	1.87, m; 1.52, m	29.0, CH_2_	1.40, m; 2.13, m
17	144.4, C		145.3, C		55.9, C		56.3, CH	1.24, m
18	16.6, CH_3_	0.84, s	16.7, CH_3_	0.88, s	12.1, CH_3_	0.65, s	12.0, CH_3_	0.64, s
19	14.0, CH_3_	0.76, s	17.5, CH_3_	0.79, s	17.2, CH_3_	0.74, s	17.5, CH_3_	1.20, s
20	124.2, C		125.4, C		36.3, CH		40.2, CH	0.84, m
21	17.9, CH_3_	1.67, s	18.1, CH_3_	1.72, s	19.1, CH_3_	0.93, d (6.6)	19.5, CH_3_	0.92, d, overlap
22	44.3, CH_2_	1.77, m; 1.87, m	45.4, CH_2_	1.80, m; 1.95, m	34.9, CH_2_	1.50, m	25.6, CH	0.31, m
23	30.2, CH	2.06, m	31.2, CH	2.10, m	28.1, CH_2_	0.77, m; 1.30, m	24.2, CH	0.53, m
24	45.6, CH	1.40, m	46.4, CH	1.46, m	45.3, CH	1.27, m	45.1, CH	0.52, m
25	74.2, C		74.5, C		73.7, C		33.0, CH	1.65, m
26	28.1, CH_3_	1.19, s	27.7, CH_3_	1.16, s	26.2, CH_3_	1.14, s	20.9, CH_3_	0.88, d (6.9)
27	28.3, CH_3_	1.19, s	28.5, CH_3_	1.18, s	27.5, CH_3_	1.16, s	18.7, CH_3_	0.85, d (6.8)
28	9.3, CH_3_	0.84, d (7.2)	9.4, CH_3_	0.85, d (7.2)	15.0, CH_3_	0.88, d (6.7)	15.9, CH_3_	0.92, d, overlap
29	15.7, CH_3_	0.81, d (6.8)	16.0, CH_3_	0.82, d (6.8)			10.7, CH_2_	0.12, m
30	16.6, CH_3_	1.00, d (6.1)						

^a^Measured at 125 MHz in CDCl_3_. ^b^Measured at 500 MHz in CDCl_3_. ^c^Measured at 125 MHz in CD_3_OD. ^d^Measured at 500 MHz in CD_3_OD.

**Figure 2:**
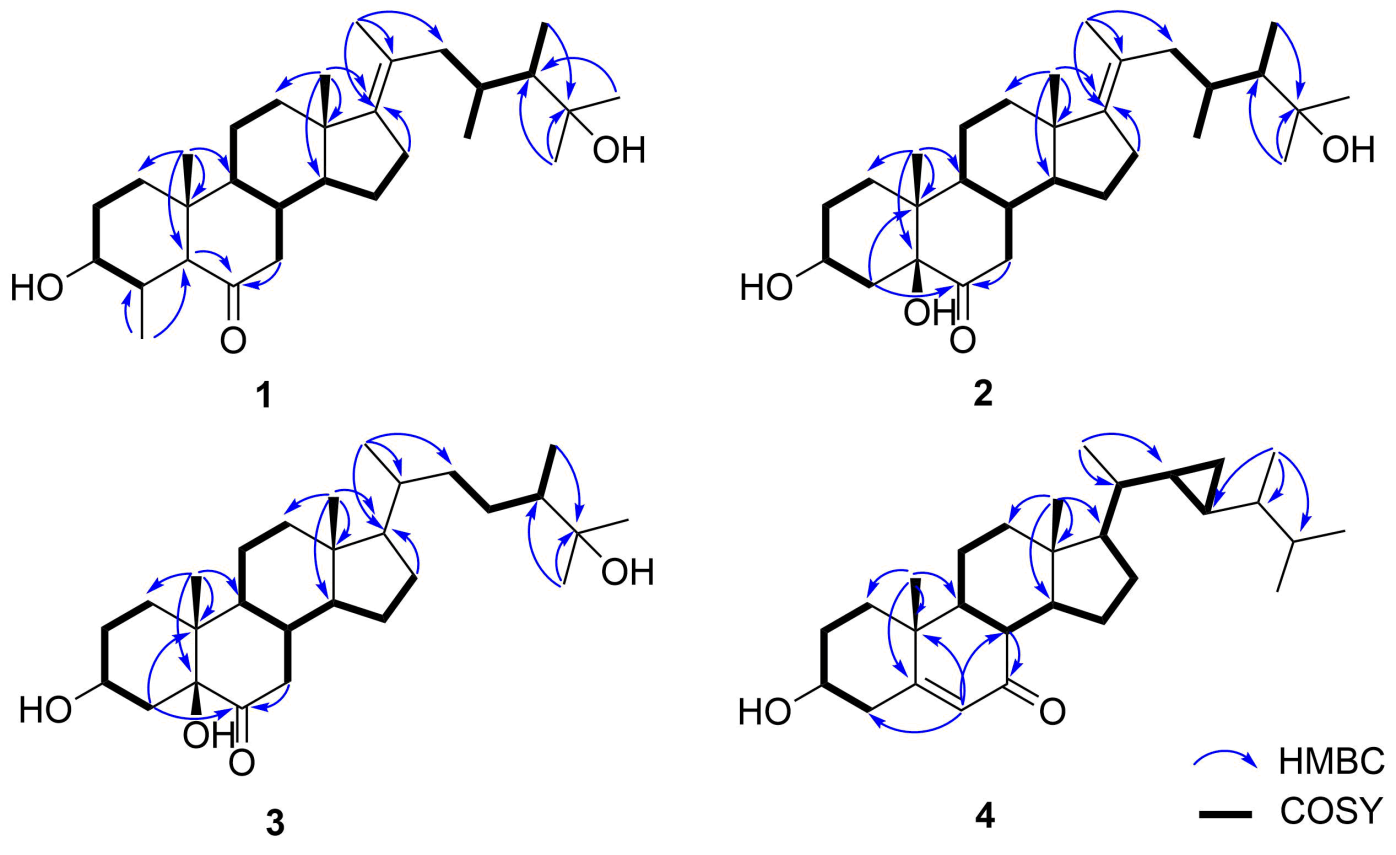
^1^H,^1^H-COSY and selected key HMBC correlations of **1**–**4**.

The ^1^H,^1^H-COSY experiment (Figure 2) revealed the proton–proton correlations of H-1/H-2/H-3/H-4, H-7/H-8/H-9/H-11/H-12, H-8/H-14/H-15/H-16, and H-22/H-23/H-24/H-28/29. These data, together with the HMBC correlations (Figure 2) from H-19 to C-1/C-5/C-9/C-10, from H_3_-30 to C-4/C-5, from H-5/H-7 to C-6, from H_3_-18 to C-12/C-13/C-14/C-17, from H_3_-21 to C-17/C-20/C-22, and from H_3_-26 to C-24/C-25 confirmed the establishment of the carbon skeleton of the 23,24-dimethycholestane with a methyl group at C-4. Thus, the planar structure of **1** was established as shown in Figure 1.

**Figure 3:**
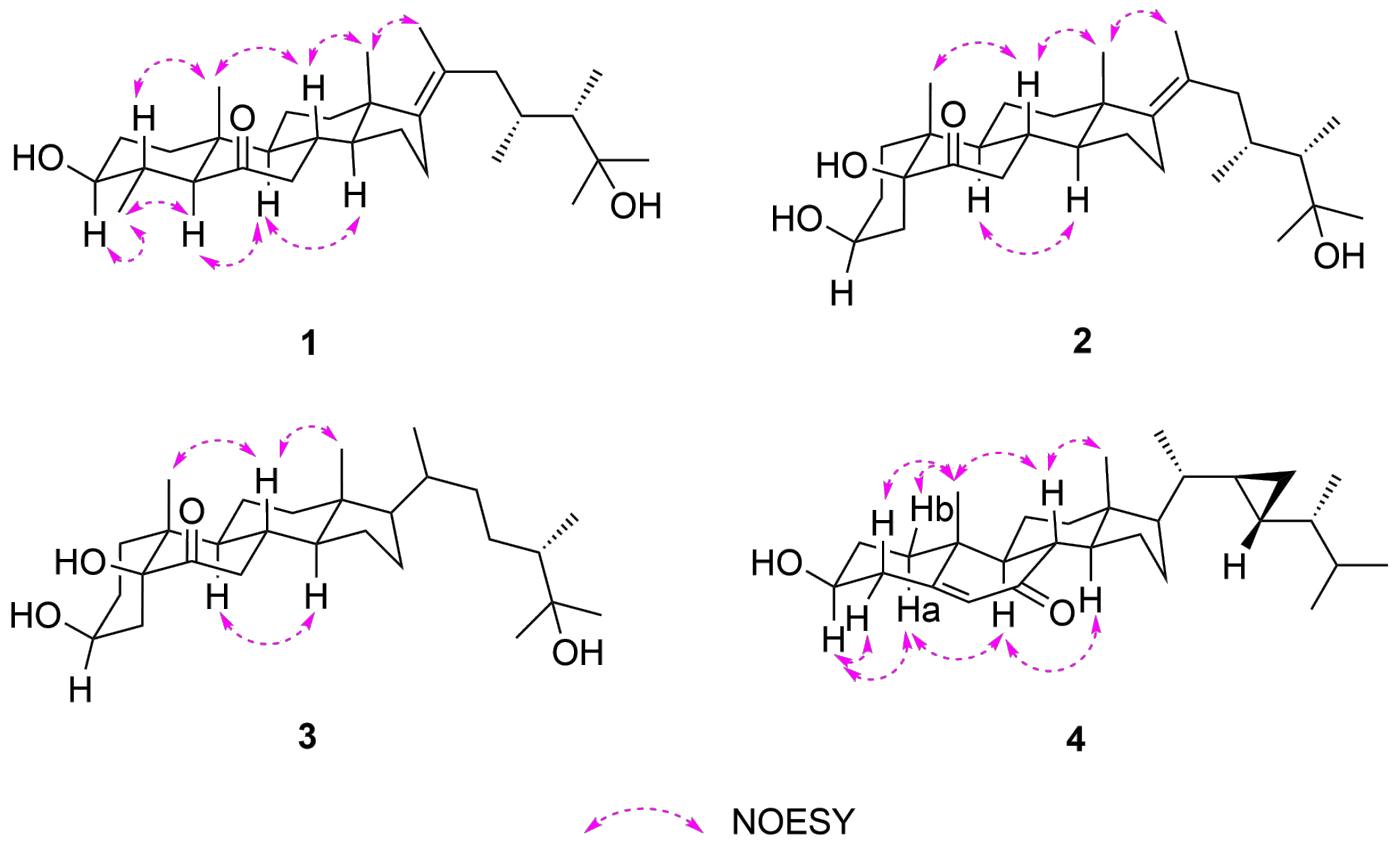
Selected NOESY correlations of compounds **1**–**4**.

**Figure 4:**
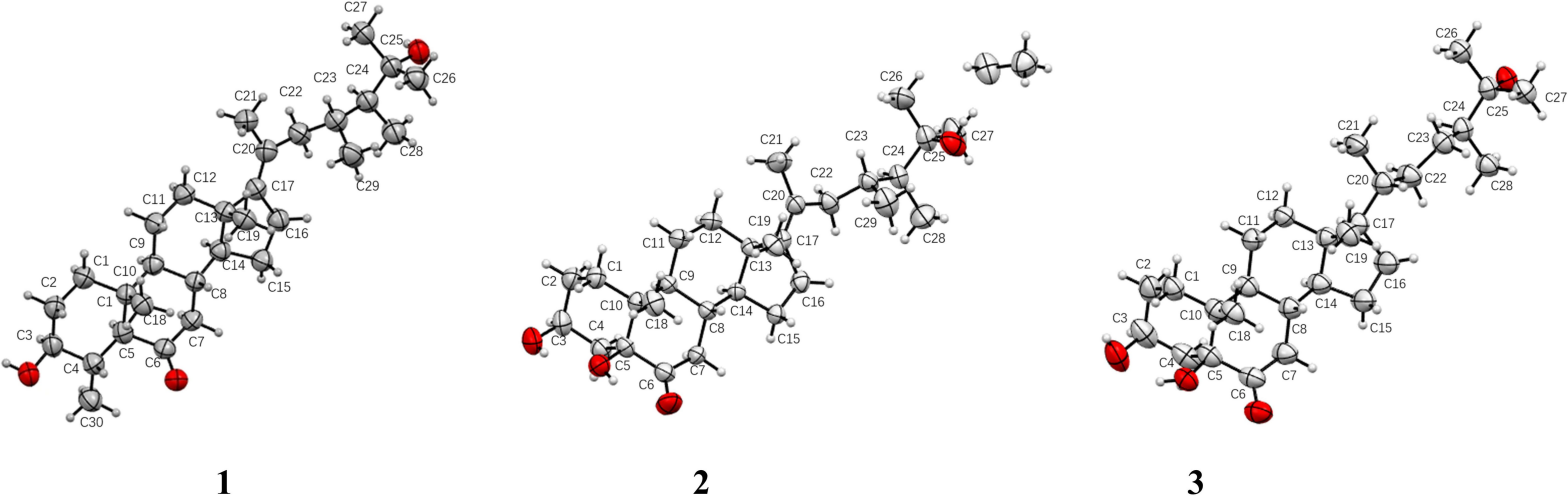
X-ray crystallographic analysis of compounds **1**–**3**.

The relative configuration of **1** was deduced by the NOESY spectrum (Figure 3). The NOESY correlations of H-4 with H_3_-19, H-8 with H_3_-18 and H_3_-19 suggested the β-orientation of H-4, H-8, H_3_-18, and H_3_-19. Moreover, H_3_-30 showed NOESY correlations with H-3/H-5, H-5 with H-9, and H-9 with H-14 indicating the α-orientation of H-3, H-5, H-9, H-14, and H_3_-30. Furthermore, the NOESY correlations of H_3_-18 with H_3_-21 suggested the *E* geometry of Δ^17(20)^. Finally, the absolute configuration of compound **1** was established by single-crystal X-ray diffraction analysis (Figure 4) carried out using Cu Kα radiation with a Flack parameter of 0.0(2).

Compound **2**, was isolated as a white powder with molecular formula C_29_H_48_O_4_, established by HREIMS at *m*/*z* 483.3446 [M + Na]^+^. The ^1^H and ^13^C NMR data (Table 1) of **2** exhibited very similar typical features of compound **1**, the difference between the two compounds occurred in ring A: an OH was located at C-5, but a missing methyl group at C-4 in **2**, which was in agreement with the ^13^C NMR spectrum and the molecular mass. The hydroxylation at C-5 was deduced from the HMBC correlations (Figure 2) from H_3_-19/H-4 to C-5. Moreover, the HMBC correlations found from H-4 to C-5/C-6, H-7 to C-6, and H_3_-18/H_3_-21 to C-17 confirmed the location of a ketone carbonyl at C-6 and the double bond at C-17, respectively. Thus, the planar structure of **2** was established as shown in Figure 1. The relative configuration of **2** was deduced by the cross-peaks shown by a 2D NOESY spectrum (Figure 3). The NOE correlations of H-8 with H_3_-18 and H_3_-19, and H-9 (δ_H_ 1.94) with H-14 (δ_H_ 1.50) indicated the β-orientation of H-8/H_3_-18/H_3_-19, while α-orientation of H-9/H-14. Furthermore, the NOESY correlation of H_3_-21 with H_3_-18 suggests the *E* geometry of ∆^17(20)^. The absolute configuration of **2** was established by single-crystal X-ray diffraction analysis (Figure 4) carried out using Cu Kα radiation with a Flack parameter of 0.3(4).

Compound **3** was isolated as a white powder with molecular formula C_28_H_48_O_4_, established by HRESIMS at *m*/*z* 449.3626 [M + H]^+^. The ^1^H and ^13^C NMR data (Table 1) of **3** were very similar to those of its analog compound **2**, showing the identical signals of the tetracyclic parent nucleus. The difference between the two compounds occurred in the side chain: A single bound was located at C-17/C-20 in **3** instead of the double bound in **2** and the absence of a methyl unit at C-23, in agreement with the ^13^C NMR spectrum and the molecular mass. The HMBC correlations (Figure 2) found from H_3_-21 to C-17/C-20/C-22, from H_3_-26 to C-25/C-27/C-24, and from H_3_-28 to C-24/C-25, together with the ^1^H,^1^H-COSY correlation (Figure 2) from H-22 to H-24 confirmed the side chain of compound **3**. Thus, the planar structure of compound **3** was established, which was the same as the known compound (3β,5α)-25-trihydroxy-24*S*-methylcholestan-6-one [8]. The difference was the configuration of C-5, which was established as *S* by single-crystal X-ray diffraction analysis (Figure 4) carried out using Cu Kα radiation with a Flack parameter of −0.11(9). Thus, the absolute configuration of **3** was established.

Compound **4** was obtained as a yellow powder. Based on the HRESIMS data (*m*/*z* 427.3569 [M + H]^+^), the molecular formula was determined to be C_29_H_46_O_2_, 14 mass units less than compound **5** [6]. By comparing the NMR data (Table 1) of **4** and **5**, it is obvious that they possess the same parent nucleus. The major difference between them was that the side chain of **4** lacks a methyl group at C-23, which can also be proved by the molecular mass. The concrete structure of the side chain was established by the COSY and HMBC correlations (Figure 2). The ^1^H,^1^H-COSY experiment revealed that the proton–proton correlation of H-17/H-20/H-21/H-22/H-23/H_2_-29. These data, together with the key HMBC correlations from H_3_-28 to C-23/C-24/C-25 and from H_3_-26/H_3_-27 to C-24/C-25, confirmed the structure of the side chain of compound **4**. The NOESY correlations (Figure 3) from H-1a (δ_H_ 1.22) to H-3 and H-9, H-9 to H-14, H-1b (δ_H_ 1.95) to H_3_-19, H-8 to H_3_-18 and H_3_-19, H_3_-18 to H-20 indicated that H-3, H-9, H-14, and H-17 were orientated on α-face, while 3-OH, H-8, H_3_-18, and H_3_-19 were positioned on the β-face. Further, the NMR data of **4** for the side chain were almost identical to the known compound **8**, demethylgorgosterol [9–10]. Thus, the structure of compound **4** was assigned as shown in Figure 1.

### Biological activity

**Figure 5:**
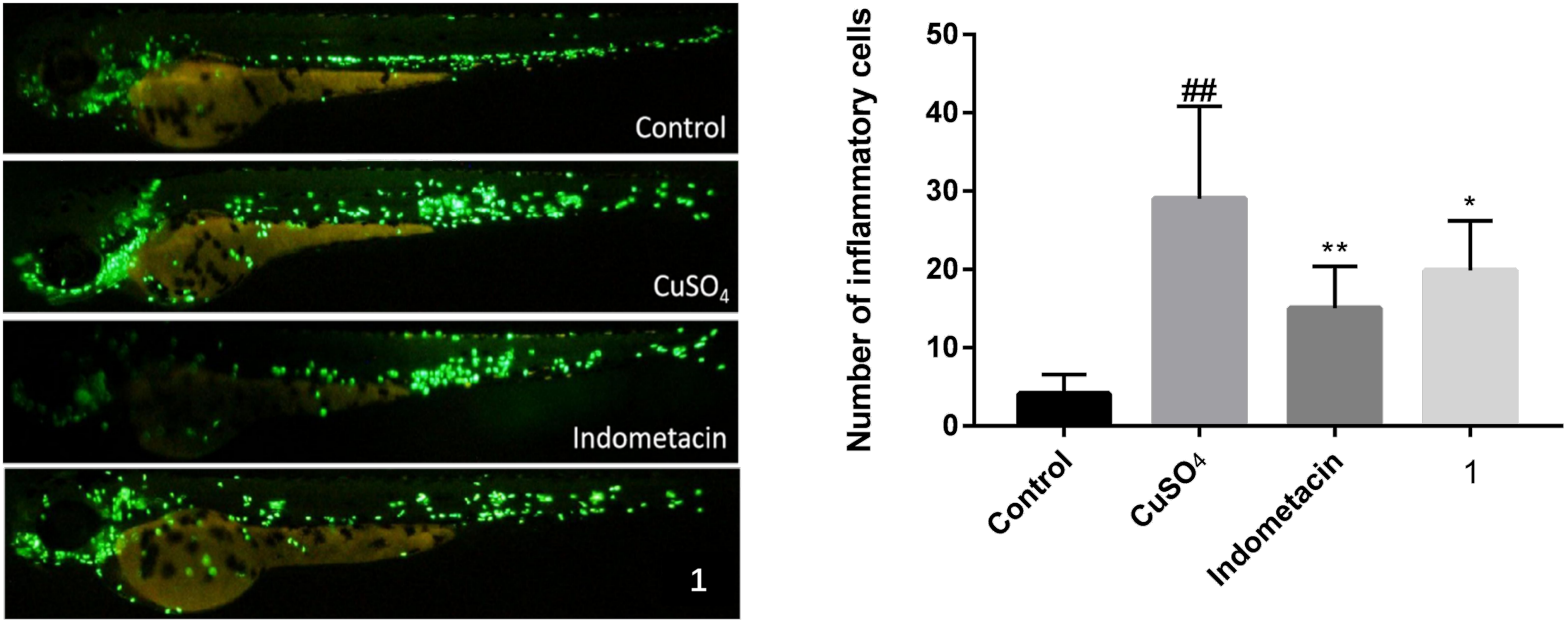
Effects of compound **1** on the anti-inflammation of zebrafish internodes. ## Indicates that the CuSO_4_ model group has a very significant difference compared with the blank group (*p* < 0.01). * and ** indicate that sample groups have significant differences compared with the CuSO_4_ model group.

The cytotoxic activities of compounds **1**–**7** were evaluated against three cancer cell lines (HL-60, K562, and Hela), but only compound **1** exhibited weak cytotoxic activity against K562 cells with an IC_50_ value of 19.03 μM. The investigation of anti-inflammatory activities of lobophysterols E–H with classic transgenic fluorescent zebrafish models (Figure 5) showed that compound **1** exhibited moderate activity, with an inhibition rate of 32% (20 μM).

## Conclusion

In conclusion, four new steroids lobophysterols E–H (**1**–**4**) and three known analogs were isolated from the soft coral *Lobophytum pauciflorum* collected at Xisha Island, China. Compounds **1** and **2** represented rare examples of steroids featuring both 23,24-dimethyl groups and 17(20)*E* double bond. Moreover, compound **1** has a tetracyclic skeleton with a methyl group at C-4. The absolute configuration of **1**–**3** was determined by X-ray diffraction analyses. Compounds **1**–**7** were subjected to a cytotoxic activity evaluation against HL-60, K562, and Hela cells, only compound **1** exhibited weak cytotoxic activity against K562 cells with an IC_50_ value of 19.03 μM. In addition, compound **1** exhibited a moderate anti-inflammatory effect in zebrafish.

## Experimental

### General experimental procedures

NMR spectra were measured via an Agilent DD2-500 spectrometer (500 MHz for ^1^H NMR and 125 MHz for ^13^C NMR). Chemical shifts are reported in parts per million (ppm) with coupling constants (*J*) in hertz relative to the solvent peaks; δ_H_ 3.31 and δ_C_ 49.0 for CD_3_OD; δ_H_ 7.26 and δ_C_ 77.16 for CDCl_3_. HRESIMS data were surveyed on a Thermo LTQ-Orbitrap mass spectrometer. IR spectra were recorded on a Nicolet NEXUS 470 spectrophotometer using KBr pellets. UV spectra were recorded on a Jasco J-815 CD spectropolarimeter. Optical rotations were measured with a Jasco P-1020 polarimeter. Semi-preparative HPLC (Agilent Technologies 1260 Infinity II) equipped with a reversed-phase column ((YMC-packed C18, 5 µm, 250 × 10 mm, 2.0 mL/min) was used to purify samples. Silica gel (300–400 mesh, Qingdao) was used for column chromatography (CC), and precoated silica gel plates (GF254, Qingdao) were used for TLC.

### Soft coral material

The soft coral *Lobophytum pauciflorum* was collected from Yongle Islands of Xisha Islands of the South China Sea in May 2012. The sample was identified by Pingjyun Sung, National Museum of Marine Biology and Aquarium (NMMBA), Checheng, Pingtung 944, Taiwan, China. The voucher specimen (No. XS-2012-27), frozen at −20 °C, was deposited at the School of Medicine and Pharmacy, Ocean University of China, P. R. China.

### Extraction and isolation

The frozen bodies of *Lobophytum pauciflorum* (3.6 kg, wet weight; 1.1 kg, dry weight) were cut into pieces and exhaustively extracted with MeOH five times at room temperature. The solvent was removed under reduced pressure and the combined organic extract was desalted three times by anhydrous methanol. The desalted residue (110 g) was subjected to silica gel column chromatography (CC) eluted with two gradient systems, PE/acetone (1:0 to 1:1) and subsequently CH_2_Cl_2_/MeOH (15:1 to 1:1) to afford 8 fractions. Fraction 4 (14.6 g) was split (chromatographed on) by silica gel eluting with a gradient of PE/acetone (30:1 to 1:1) to give three subfractions (F41–F43). Subfraction F41 (2.1 g) was chromatographed over silica gel column (PE/acetone, 50:1 to 2:1) to give seven subfractions (F411–F417), F411 (330 mg) was purified by semi-preparative HPLC (ODS, 5 µm, 250 × 10 mm; methanol/water, 95:5, v/v; 2.0 mL/min) to afford compound **4** (2.3 mg), **5** (4.7 mg), **6** (15.5 mg) and **7** (10.2 mg). Fr.412 (210 mg) was chromatographed on semi-preparative HPLC (ODS, 5 µm, 250 × 10 mm; methanol/water, 85:15, v/v; 2.0 mL/min) to give compound **1** (2.5 mg), **2** (5.8 mg) and **3** (13.3 mg).

### Identification of new compounds

**Compound 1**: colorless crystals; [α]_D_^25^ −19.28 (*c* 0.13, MeOH); IR (KBr) ν_max_: 3389, 2944, 2871, 1707, 1603, 1467, 1380 cm^−1^; ^1^H and ^13^C NMR data (CDCl_3_, 500 and 125 MHz) see Table 1; HRESIMS (*m*/*z*) [M + Na]^+^ calcd for C_30_H_50_O_3_Na, 481.3652; found, 481.3648.

**Compound 2**: colorless crystals; [α]_D_^25^ −12.77 (*c* 0.3, MeOH); IR (KBr) ν_max_: 3361, 2926, 2855, 1702, 1651, 1459, 1376 cm^−1^; ^1^H and ^13^C NMR data (CD_3_OD, 500 and 125 MHz) see Table 1; HRESIMS (*m*/*z*) [M + Na]^+^ calcd for C_29_H_48_O_4_Na, 483.3445; found, 483.3446.

**Compound 3**: colorless crystals; [α]_D_^25^ −18.36 (*c* 0.5, MeOH); IR (KBr) ν_max_: 3390, 2938, 1702, 1459, 1376 cm^−1^; ^1^H and ^13^C NMR data (CDCl_3_, 500 and 125 MHz) see Table 1; HRESIMS (*m*/*z*) [M + H]^+^ calcd for C_28_H_49_O_4_, 449.3625; found, 449.3626.

**Compound 4**: yelllow crystals; [α]_D_^25^ −27.83 (*c* 0.13, MeOH); IR (KBr) ν_max_: 3391, 2957, 2872, 1683, 1650, 1558, 1540, 1357 cm^−1^; ^1^H and ^13^C NMR data (CDCl_3_, 500 and 125 MHz) see Table 1; HRESIMS (*m*/*z*) [M + H]^+^ calcd for C_29_H_47_O_2_, 427.3571; found, 427.3569.

### Cytotoxicity assays

In vitro cytotoxicity was determined by the MTT method against K562 (chronic myeloid leukemia) and HL-60 (human promyelocytic leukemia) cell lines, and by the SRB method against the Hela cell line.

### Zebrafish maintenance

Adult zebrafish were cultivated by Qilu University of Technology (Jinan, China). Transgenic zebrafish [Tg: zlyz-EGFP and Tg (vegfr2: GFP)] expressing enhanced green fluorescent protein (EGFP) was used in this article. The conditions of the maintenance complied with guidelines of the Organization for Economic Co-operation and Development (OECD). The zebrafish were maintained under a 14/10 h light/dark cycle at the temperature (28 ± 0.5°C) in a closed flow-through system with charcoal-filtered tap water to ensure normal spawning.

### CuSO_4_-induced model of zebrafish

In a manner similar to literature reference [11], healthy zebrafish larvae were selected into 24-well plates (*n* = 10/well) in a 2 mL final volume of embryo medium at 3 dpf and divided into five groups: a control group (fresh fish water), a model group: 40 μM CuSO_4_, a positive drug group: 50 μM indomethacin (Solarbio, China) and drug groups: 20 μM CuSO_4_ (Sigma-Aldrich, St. Louis, MO, USA) was added to the drug groups and incubated for 1 h after treatment with different compounds for 2 h. All treatments were performed in triplicate. Each zebrafish larva was photographed by a fluorescence microscope (AXIO, Zoom. V16), and the number of macrophages was counted by using the Image-Pro Plus software.

## Supporting Information

File 1 Crystal data and structure refinement for compounds **1**–**3** and NMR, MS, and IR spectra of compounds **1**–**4**.
