# Supplementary material for: Four bioactive new steroids from the soft coral Lobophytum pauciflorum collected in South China Sea
Source: Beilstein J Org Chem. 2022 Apr 8;18:374–80. doi: 10.3762/bjoc.18.42 (PMC9016341; doi:10.3762/bjoc.18.42)
Supplement: File 1 — Crystal data and structure refinement for compounds 1–3 and NMR, MS, and IR spectra of compounds 1–4. [file Beilstein_J_Org_Chem-18-374-s001.pdf]

BEILSTEIN JOURNAL OF ORGANIC CHEMISTRY

Supporting Information

for

Four bioactive new steroids from the soft coral *Lobophytum pauciflorum* collected in South China Sea

Di Zhang, Zhe Wang, Xiao Han, Xiao-Lei Li, Zhong-Yu Lu, Bei-Bei Dou, Wen-Ze Zhang, Xu-Li Tang, Ping-Lin Li and Guo-Qiang Li

Beilstein J. Org. Chem. **2022,** *18,* 374–380. doi:10.3762/bjoc.18.42

Crystal data and structure refinement for compounds 1–3 and NMR, MS, and IR spectra of compounds 1–4

License and Terms: This is a supporting information file under the terms of the Creative Commons Attribution License (https://creativecommons.org/licenses/by/4.0). Please note that the reuse, redistribution and reproduction in particular requires that the author(s) and source are credited and that individual graphics may be subject to special legal provisions.
The license is subject to the *Beilstein Journal of Organic Chemistry* terms and conditions: (https://www.beilstein-journals.org/bjoc/terms)

Content

Table S1. Crystal data and structure refinement for compound **1**.......... S4
Table S2. Crystal data and structure refinement for compound **2**.......... S5
Table S3. Crystal data and structure refinement for compound **3**.......... S6
Fig.S1. HRESIMS spectrum of compound **1**................................ S7
Fig.S2. ^{1}H NMR spectrum of compound **1** in $CDCl_3$.......................... S7
Fig.S3. The amplificatory ^{1}H NMR spectrum of compound **1** in $CDCl_3$. S8
Fig.S4. ^{13}C NMR spectrum of compound **1** in $CDCl_3$. S8
Fig.S5. The amplificatory ^{13}C NMR spectrum of compound **1** in $CDCl_3$......... S9
Fig.S6. DEPT spectrum of compound **1** in $CDCl_3$............................ S9
Fig.S7. ^{1}H,^{1}H-COSY spectrum of compound **1** in $CDCl_3$..................... S10
Fig.S8. HSQC spectrum of compound **1** in $CDCl_3$........................... S10
Fig.S9. HMBC spectrum of compound **1** in $CDCl_3$........................... S11
Fig.S10. NOESY spectrum of compound **1** in $CDCl_3$......................... S11
Fig.S11. HRESIMS spectrum of compound **2**. S12
Fig.S12. ^{1}H NMR spectrum of compound **2** in CD_3OD........................ S12
Fig.S13. The amplificatory ^{1}H NMR spectrum of compound **2** in CD_3OD. S13
Fig.S14. ^{13}C NMR spectrum of compound **2** in CD_3OD. S13
Fig.S15. The amplificatory ^{13}C NMR spectrum of compound **2** in CD_3OD. S14
Fig.S16. DEPT spectrum of compound **2** in CD_3OD.......................... S14
Fig.S17. HSQC spectrum of compound **2** in CD_3OD.......................... S15
Fig.S18. ^{1}H,^{1}H COSY spectrum of compound **2** in CD_3OD..................... S15
Fig.S19. HMBC spectrum of compound **2** in CD_3OD.......................... S16
Fig.S20. NOESY spectrum of compound **2** in CD_3OD......................... S16
Fig.S21. ^{1}H NMR spectrum of compound **2** in $CDCl_3$. S17
Fig.S22. ^{13}C NMR spectrum of compound **2** in $CDCl_3$....................... S17
Fig.S23. HRESIMS spectrum of compound **3**. S18
Fig.S24. ^{1}H NMR spectrum of compound **3** in $CDCl_3$........................ S18
Fig.S25. The amplificatory ^{1}H NMR spectrum of compound **3** in $CDCl_3$....... S19
Fig.S26. ^{13}C NMR spectrum of compound **3** in $CDCl_3$....................... S19
Fig.S27. The amplificatory ^{13}C NMR spectrum of compound **3** in $CDCl_3$....... S20
Fig.S28. DEPT spectrum of compound **3** in $CDCl_3$.......................... S20
Fig.S29. HSQC spectrum of compound **3** in $CDCl_3$.......................... S21
Fig.S30. ^{1}H,^{1}H-COSY spectrum of compound **3** in $CDCl_3$.................... S21
Fig.S31. HMBC spectrum of compound **3** in $CDCl_3$.......................... S22
Fig.S32. NOESY spectrum of compound **3** in $CDCl_3$......................... S22
Fig.S33. HRESIMS spectrum of compound **4**. S23

Fig.S34. ^{1}H NMR spectrum of compound **4** in $CDCl_3$........................ S23
Fig.S35. The amplificatory ^{1}H NMR spectrum of compound **4** in $CDCl_3$....... S24
Fig.S36. ^{13}C NMR spectrum of compound **4** in $CDCl_3$....................... S24
Fig.S37. The amplificatory ^{13}C NMR spectrum of compound **4** in $CDCl_3$....... S25
Fig.S38. DEPT spectrum of compound **4** in $CDCl_3$......................... S25
Fig.S39. HSQC spectrum of compound **4** in $CDCl_3$......................... S26
Fig. S40. ^{1}H,^{1}H-COSY spectrum of compound **4** in $CDCl_3$................... S26
Fig. S41. HMBC spectrum of compound **4** in $CDCl_3$........................ S27
Fig. S42. NOESY spectrum of compound **4** in $CDCl_3$....................... S27
Fig. S43. IR (KBr disc) spectrum of compound **1**. S28
Fig. S44. IR (KBr disc) spectrum of compound **2**. S28
Fig. S45. IR (KBr disc) spectrum of compound **3**. S28
Fig. S46. IR (KBr disc) spectrum of compound **4**. S29

Table S1. Crystal data and structure refinement for compound **1**.

Identification code	cu_0717_4_0m
Empirical formula	$C_{60}H_{102}O_7$
Formula weight	935.41
Temperature/K	150.0
Crystal system	triclinic
Space group	P1
a/Å	7.5758(4)
b/Å	19.0127(11)
c/Å	20.3233(11)
α/°	69.925(3)
β/°	89.856(3)
γ/°	79.736(3)
Volume/Å^3	2700.0(3)
Z	2
ρ_{calc}g/cm^3	1.151
μ/mm^{-1}	0.562
F(000)	1036.0
Crystal size/mm^3	0.11 × 0.1 × 0.09
Radiation	Cu Kα (λ = 1.54178)
Index ranges	$-8 \leq h \leq 8$, $-22 \leq k \leq 22$, $-24 \leq l \leq 23$
Reflections collected	32954
Independent reflections	12597 [R_{int} = 0.0693, R_{sigma} = 0.0843]
Data/restraints/parameters	12597/15/1287
Goodness-of-fit on F^2	1.060
Final R indexes [I>=2σ (I)]	R_1 = 0.0659, wR_2 = 0.1674
Final R indexes [all data]	R_1 = 0.0747, wR_2 = 0.1788
Largest diff. peak/hole / e Å^{-3}	0.23/-0.20
Flack parameter	0.0(2)

Crystallographic data of **1** have been deposited in the Cambridge Crystallographic Data Center (Deposition number: CCDC 2133384). The data can be obtained free of charge via www.ccdc.cam.ac.uk/products/csd/request.

Table S2. Crystal data and structure refinement for compound **2**.

Empirical formula	$C_{30}H_{52}O_5$
Formula weight	492.72
Temperature/K	293
Crystal system	monoclinic
Space group	P21
a/Å	8.8931(14)
b/Å	10.1189(16)
c/Å	16.102(2)
α/°	90
β/°	96.085(13)
γ/°	90
Volume/Å^3	1440.8(4)
Z	2
ρ_{calc}g/cm^3	1.136
μ/mm^{-1}	0.590
F(000)	544
Crystal size/mm^3	0.12 × 0.12 × 0.11
Radiation	Cu Kα (λ = 1.54184)
Index ranges	$-10 \leq h \leq 10$, $-12 \leq k \leq 12$, $-19 \leq l \leq 19$
Reflections collected	4834
Independent reflections	3381 [R_{int} = 0.0424, R_{sigma} = 0.0812]
Data/restraints/parameters	3381/1/329
Goodness-of-fit on F^2	0.974
Final R indexes [I>=2σ (I)]	R_1 = 0.0532, wR_2 = 0.0957
Final R indexes [all data]	R_1 = 0.096, wR_2 = 0.1190
Largest diff. peak/hole / e Å^{-3}	0.16/-0.134
Flack parameter	0.3(4)

Crystallographic data of **2** have been deposited in the Cambridge Crystallographic Data Center (Deposition number: CCDC 2133391). The data can be obtained free of charge via www.ccdc.cam.ac.uk/products/csd/request.

Table S3. Crystal data and structure refinement for compound **3**.

Empirical formula	$C_{28}H_{48}O_4$
Formula weight	448.66
Temperature/K	150.0
Crystal system	orthorhombic
Space group	$P2_12_12$
a/Å	10.3615(3)
b/Å	33.2315(8)
c/Å	7.4685(2)
α/°	90
β/°	90
γ/°	90
Volume/Å^3	2571.61(12)
Z	4
ρ_{calc}g/cm^3	1.159
μ/mm^{-1}	0.586
F(000)	992.0
Crystal size/mm^3	0.12 × 0.1 × 0.09
Radiation	Cu Kα (λ = 1.54178)
Index ranges	$-12 \leq h \leq 12$, $-39 \leq k \leq 39$, $-8 \leq l \leq 8$
Reflections collected	23207
Independent reflections	4477 [R_{int} = 0.0407, R_{sigma} = 0.0259]
Data/restraints/parameters	4477/0/295
Goodness-of-fit on F^2	1.074
Final R indexes [I>=2σ (I)]	R_1 = 0.0600, wR_2 = 0.1521
Final R indexes [all data]	R_1 = 0.0646, wR_2 = 0.1583
Largest diff. peak/hole / e Å^{-3}	0.27/-0.18
Flack parameter	-0.11(9)

Crystallographic data of **3** have been deposited in the Cambridge Crystallographic Data Center (Deposition number: CCDC 2133404). The data can be obtained free of charge via www.ccdc.cam.ac.uk/products/csd/request.

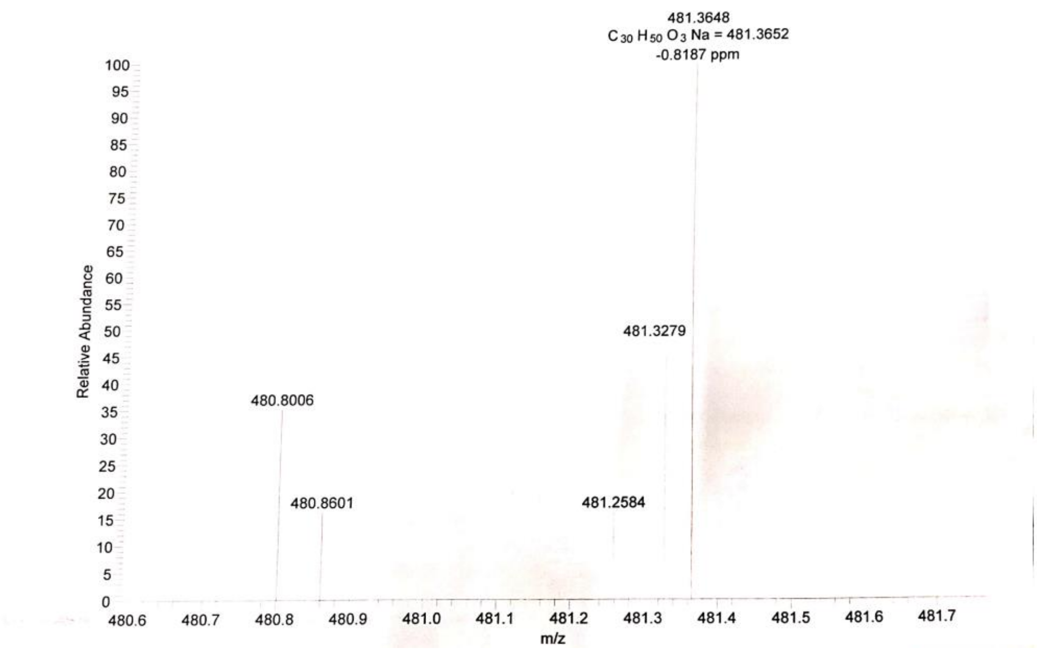

Fig.S1. HRESIMS spectrum of compound **1**.

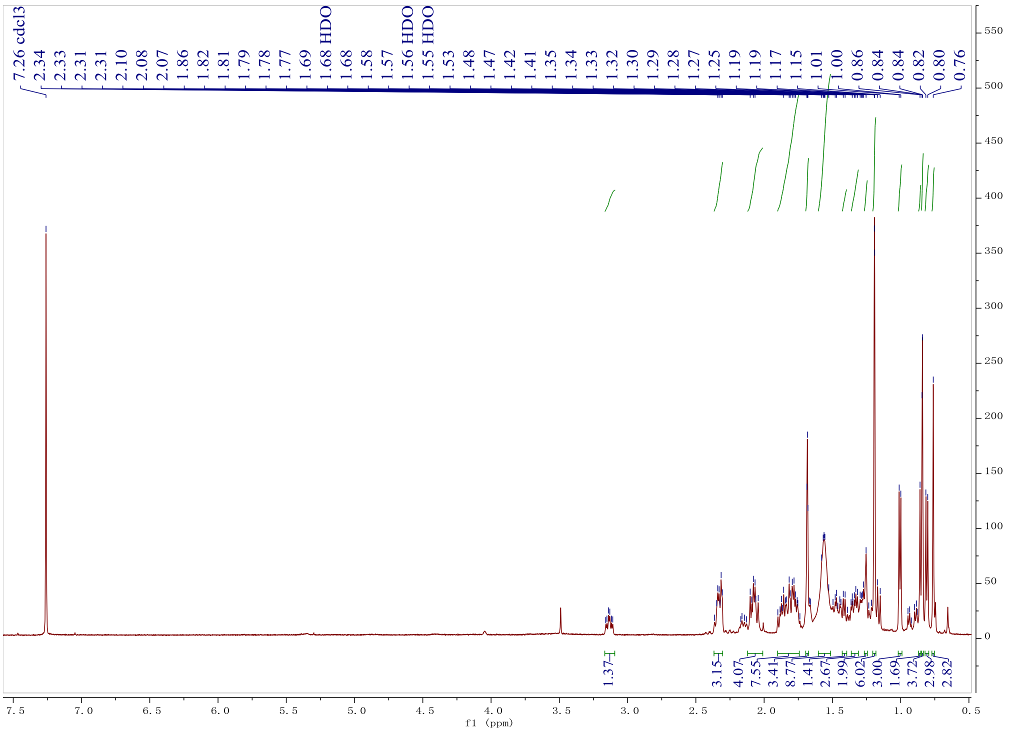

Fig.S2. ^{1}H NMR spectrum of compound **1** in $CDCl_3$.

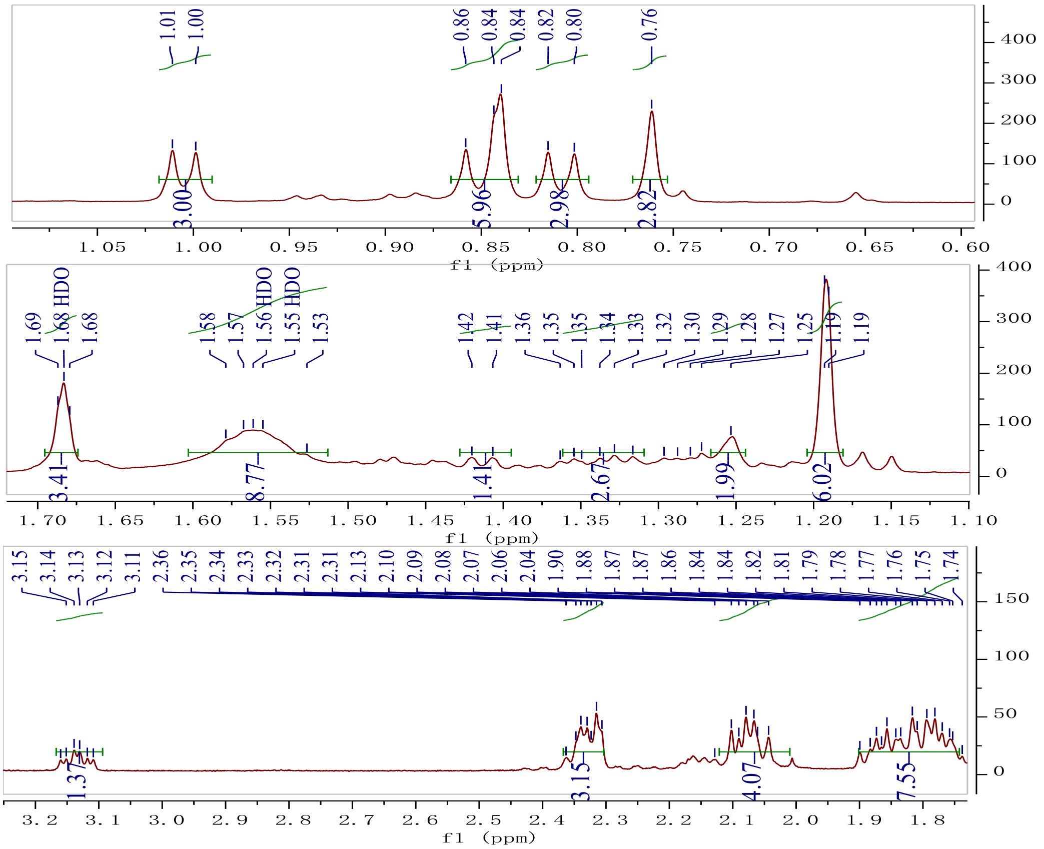

Fig.S3. The amplificatory ^{1}H NMR spectrum of compound **1** in $CDCl_3$.

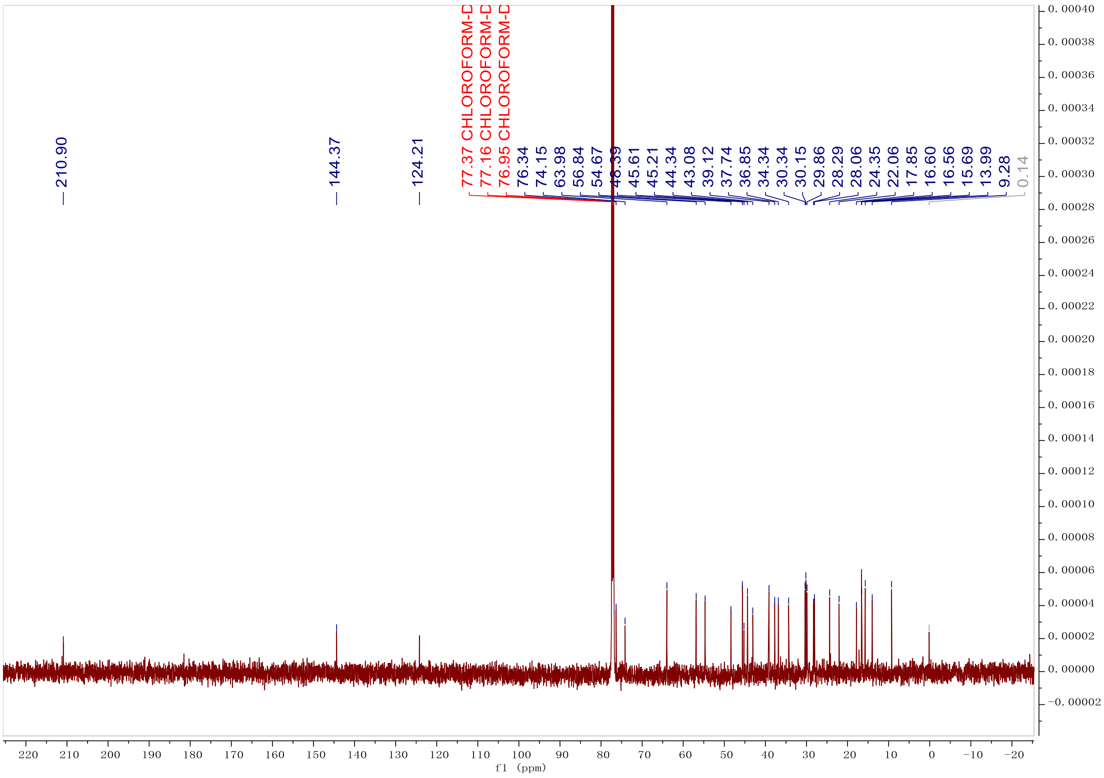

Fig.S4. ^{13}C NMR spectrum of compound **1** in $CDCl_3$.

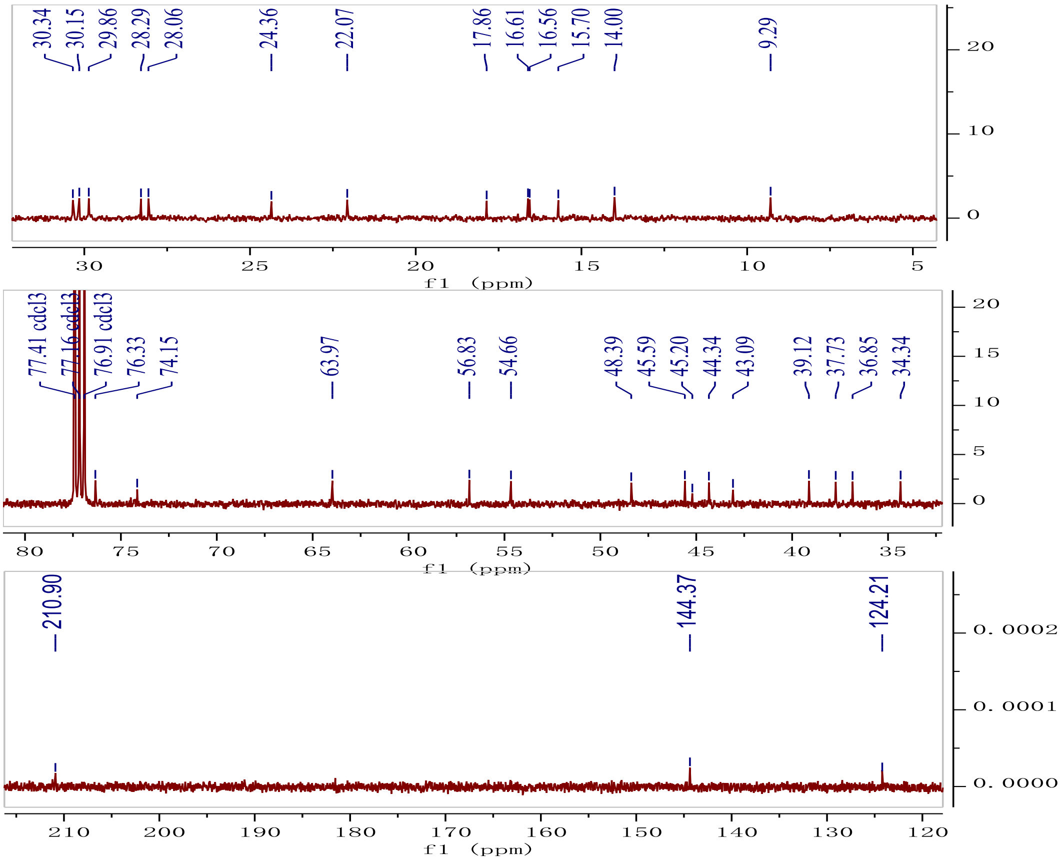

Fig.S5. The amplificatory ^{13}C NMR spectrum of compound **1** in $CDCl_3$.

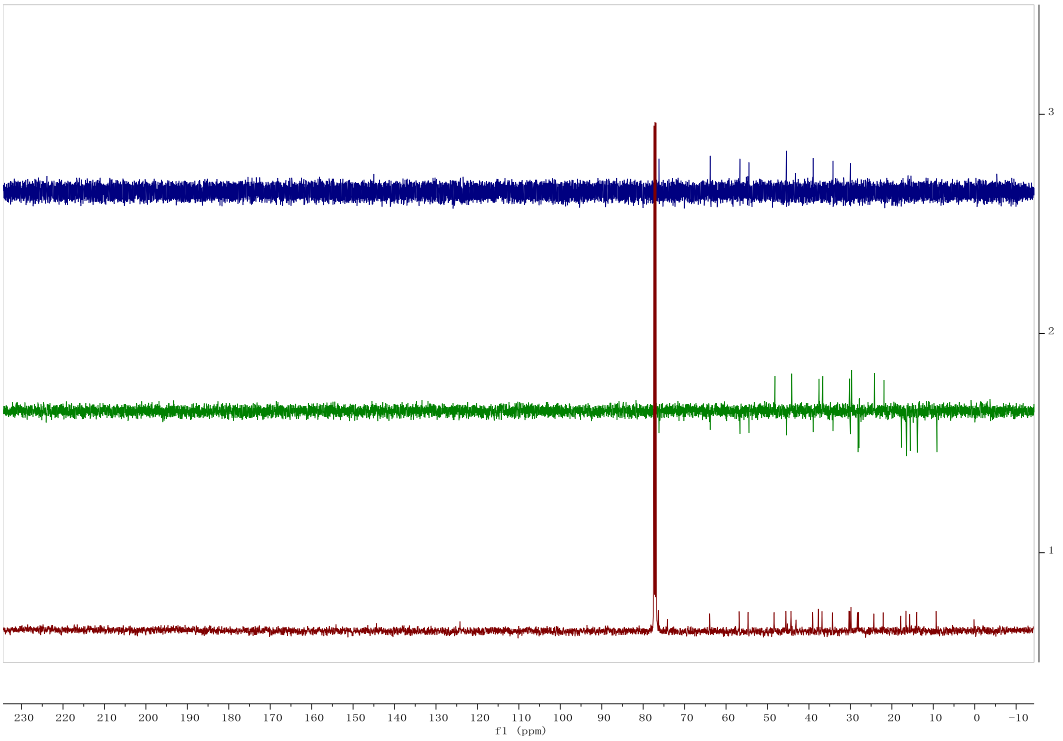

Fig.S6. DEPT spectrum of compound **1** in $CDCl_3$.

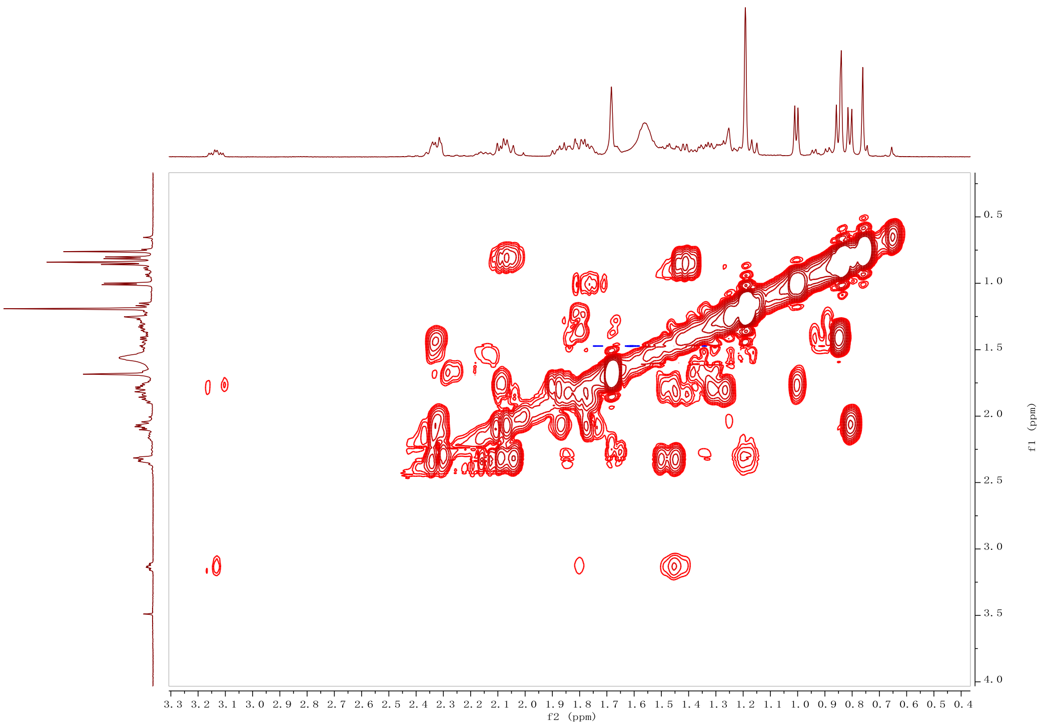

Fig.S7. ^{1}H,^{1}H-COSY spectrum of compound **1** in $CDCl_3$.

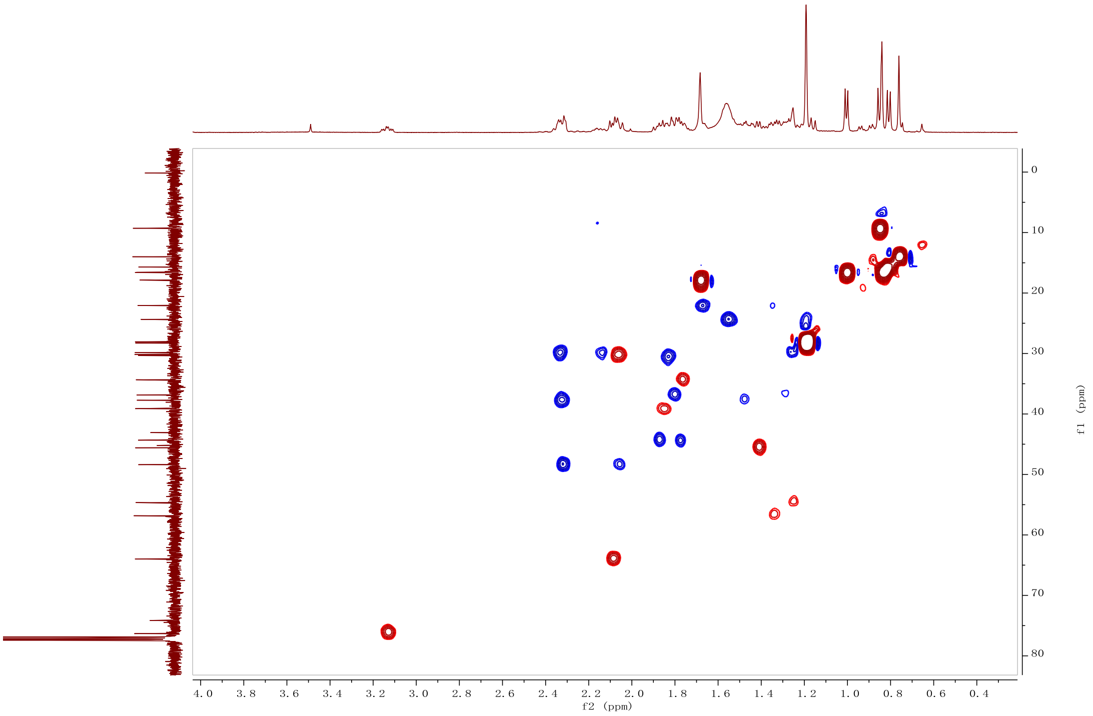

Fig.S8. HSQC spectrum of compound **1** in $CDCl_3$.

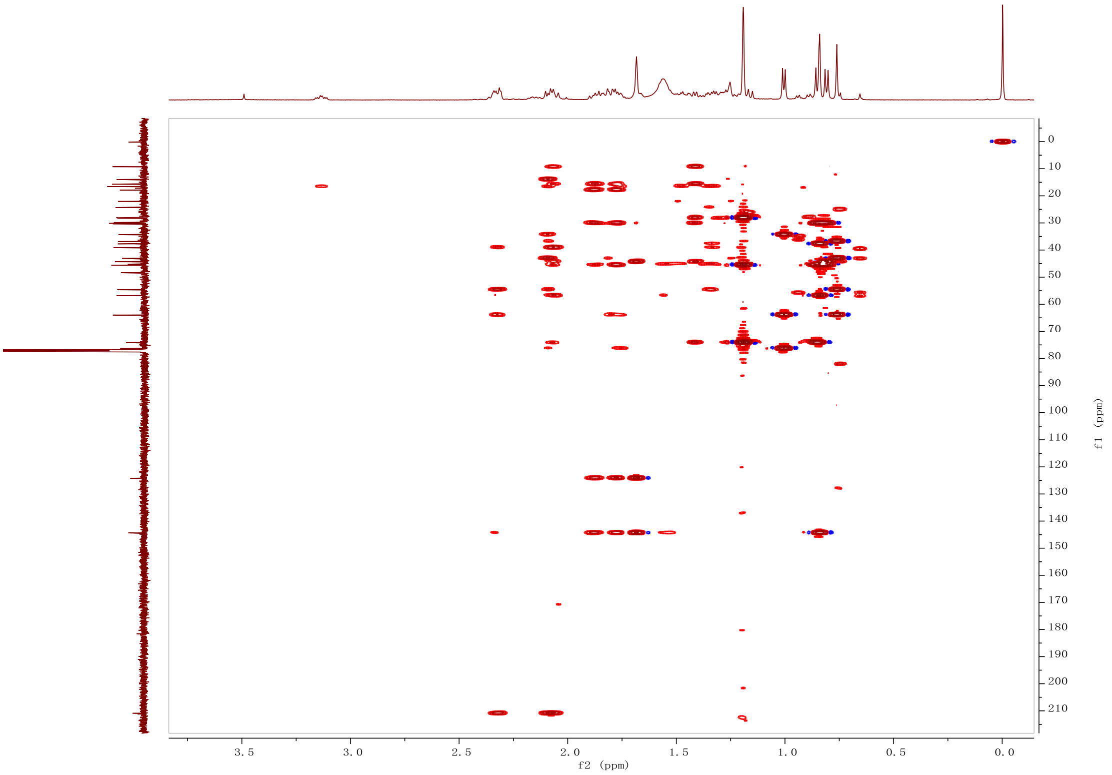

Fig.S9. HMBC spectrum of compound **1** in $CDCl_3$.

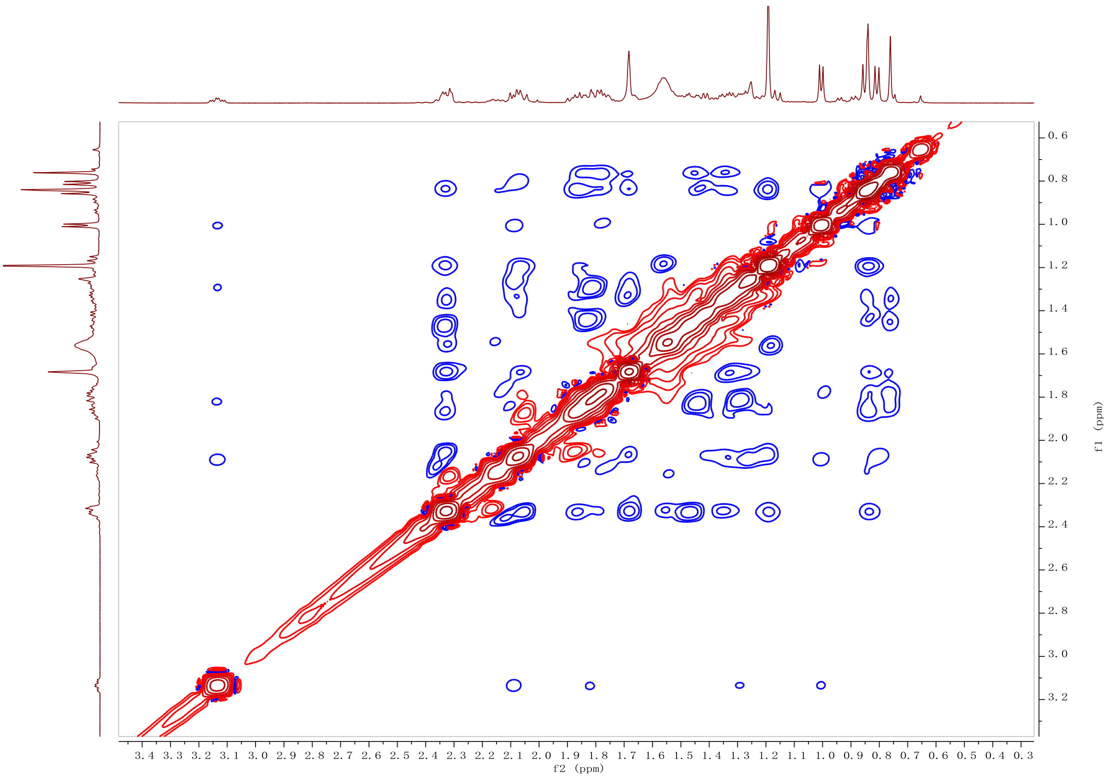

Fig.S10. NOESY spectrum of compound **1** in $CDCl_3$.

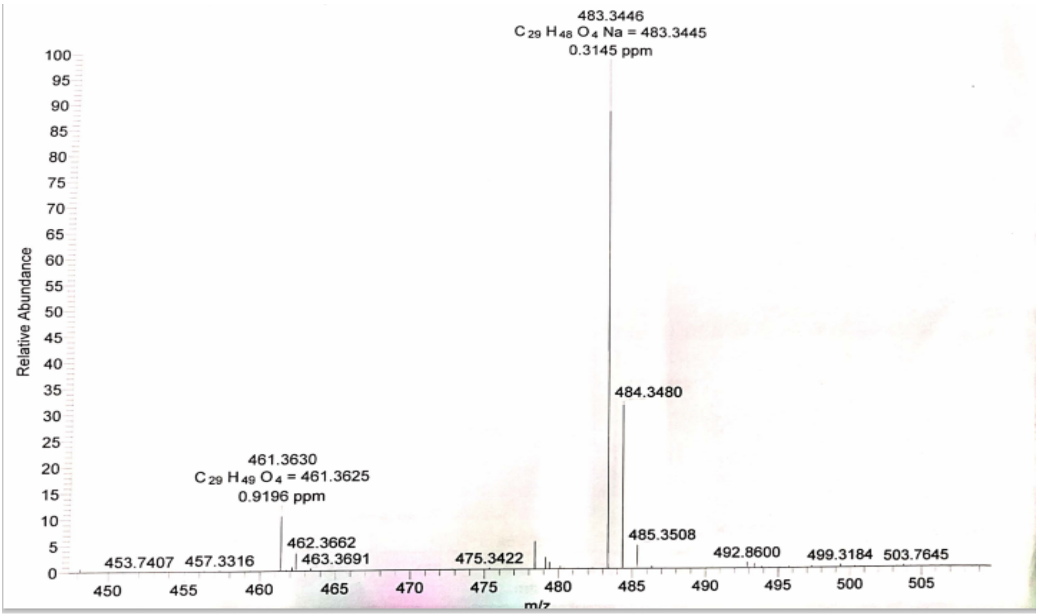

Fig.S11. HRESIMS spectrum of compound **2**.

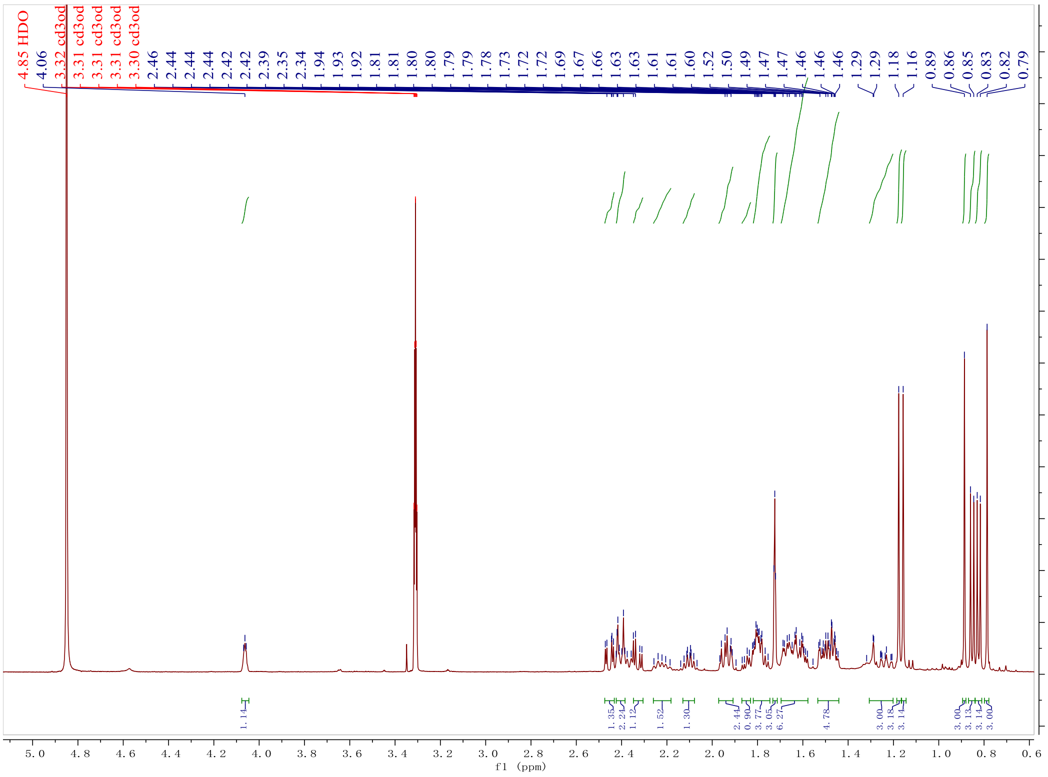

Fig.S12. ^{1}H NMR spectrum of compound **2** in CD_3OD.

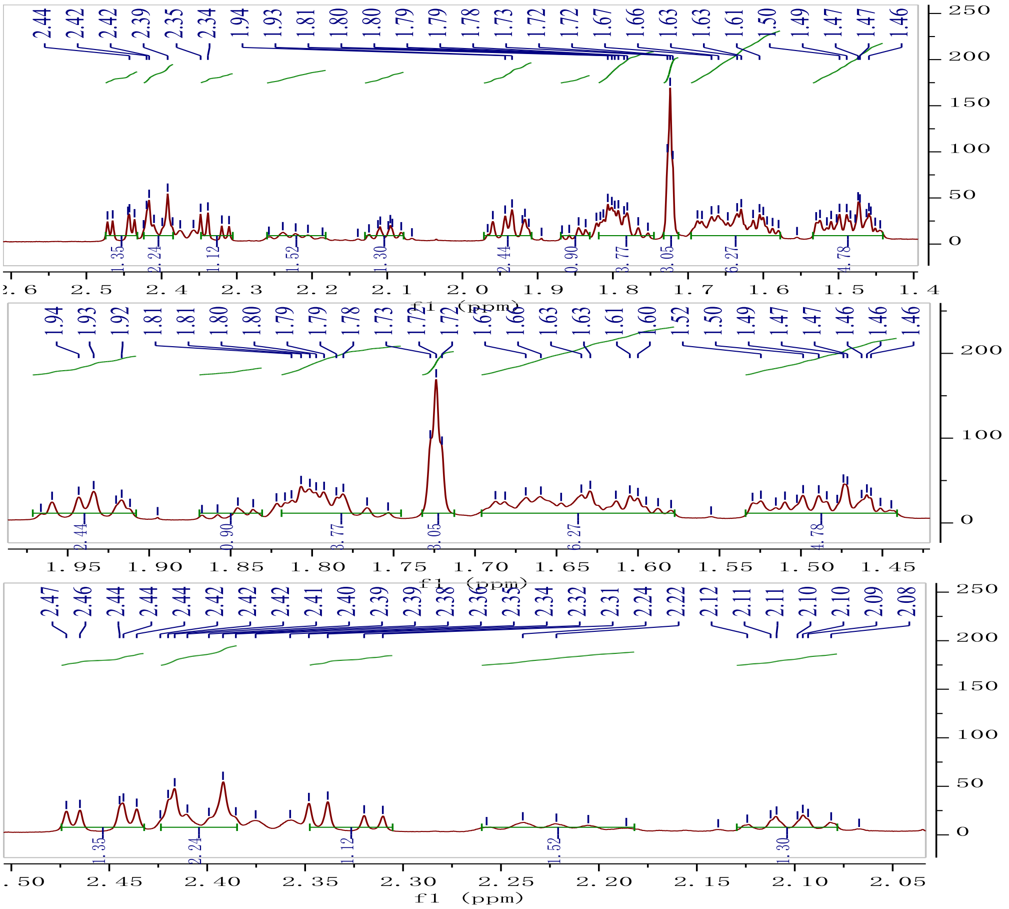

Fig.S13. The amplificatory ^{1}H NMR spectrum of compound **2** in CD_3OD.

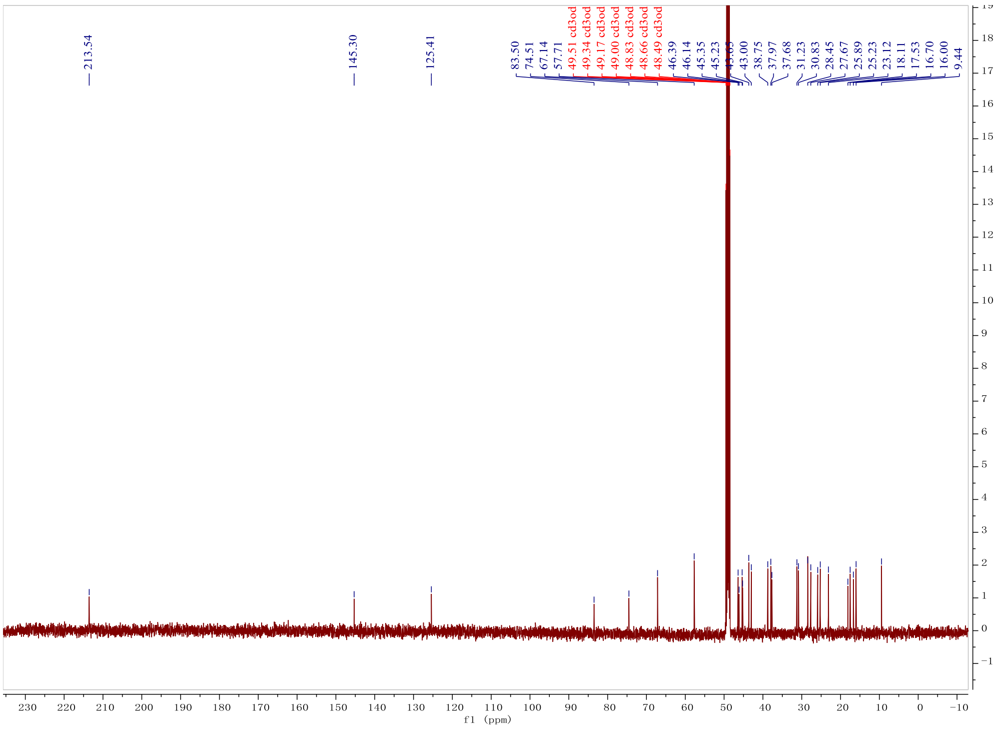

Fig.S14. ^{13}C NMR spectrum of compound **2** in CD_3OD.

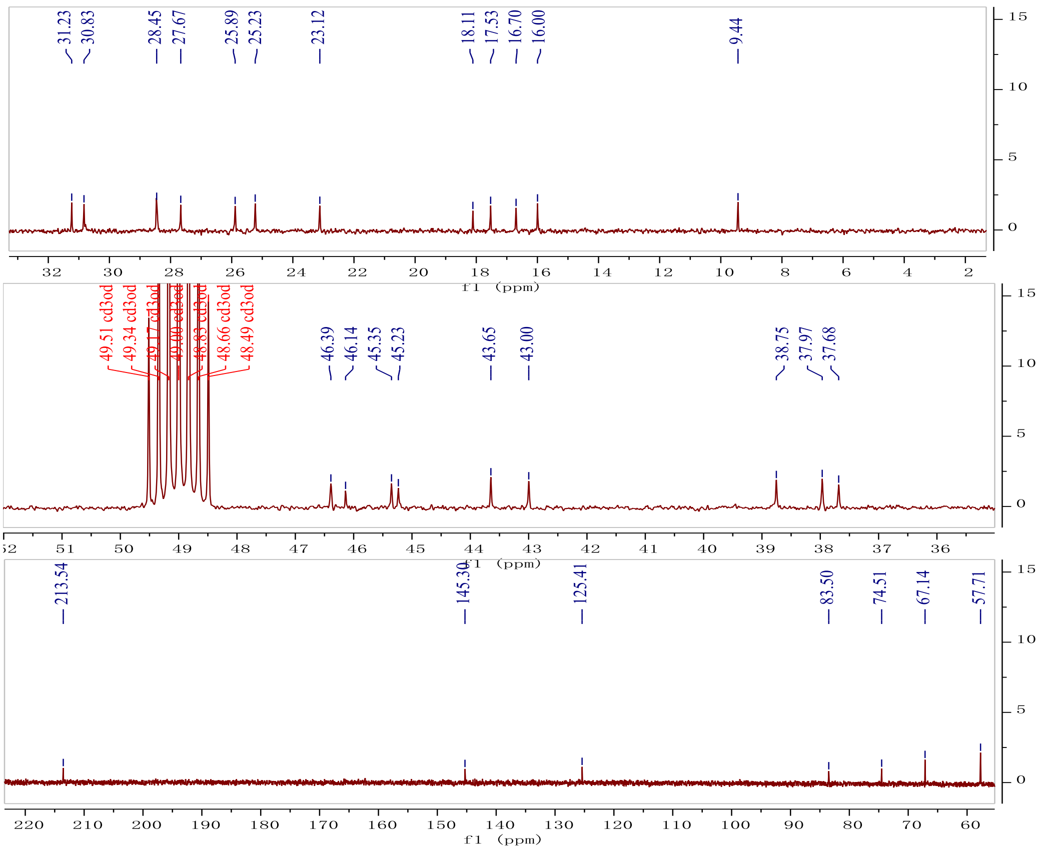

Fig.S15. The amplificatory ^{13}C NMR spectrum of compound **2** in CD_3OD.

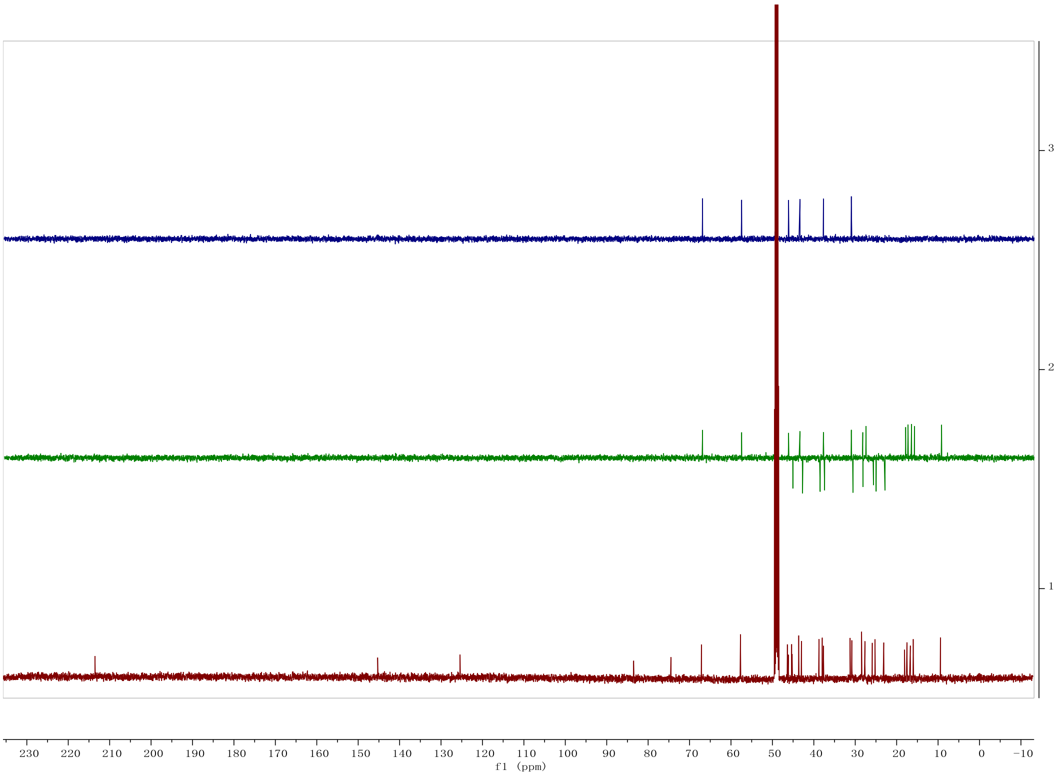

Fig.S16. DEPT spectrum of compound **2** in CD_3OD.

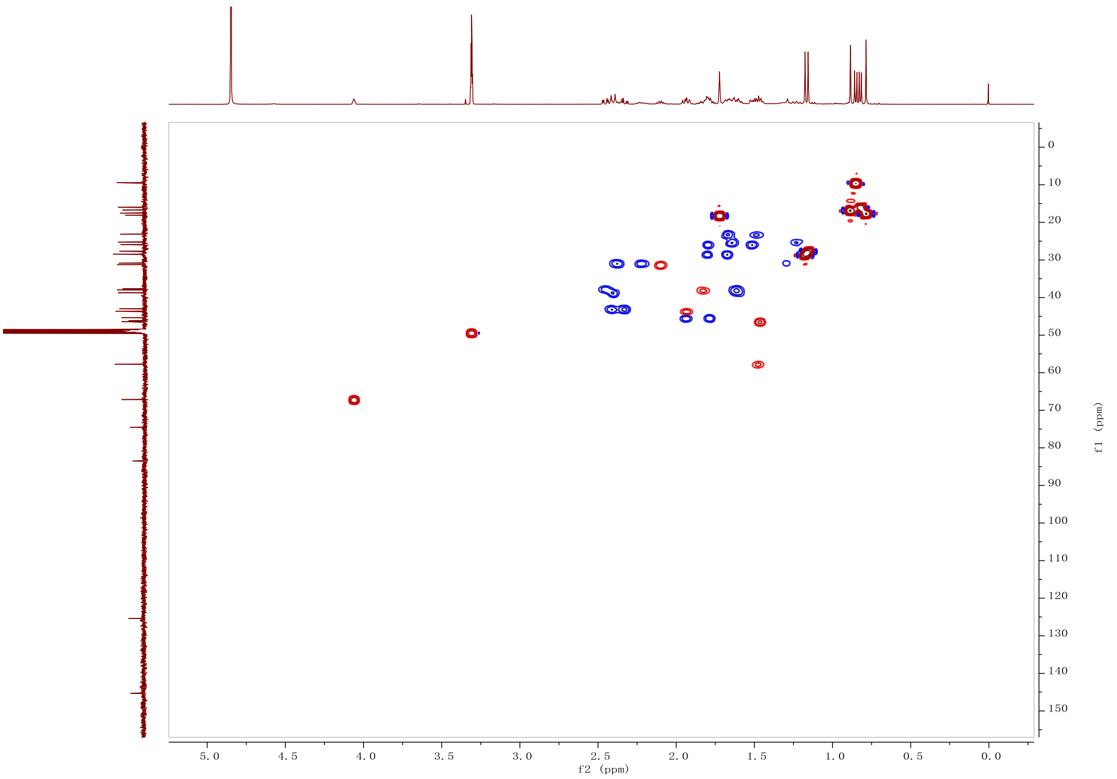

Fig.S17. HSQC spectrum of compound **2** in CD3OD.

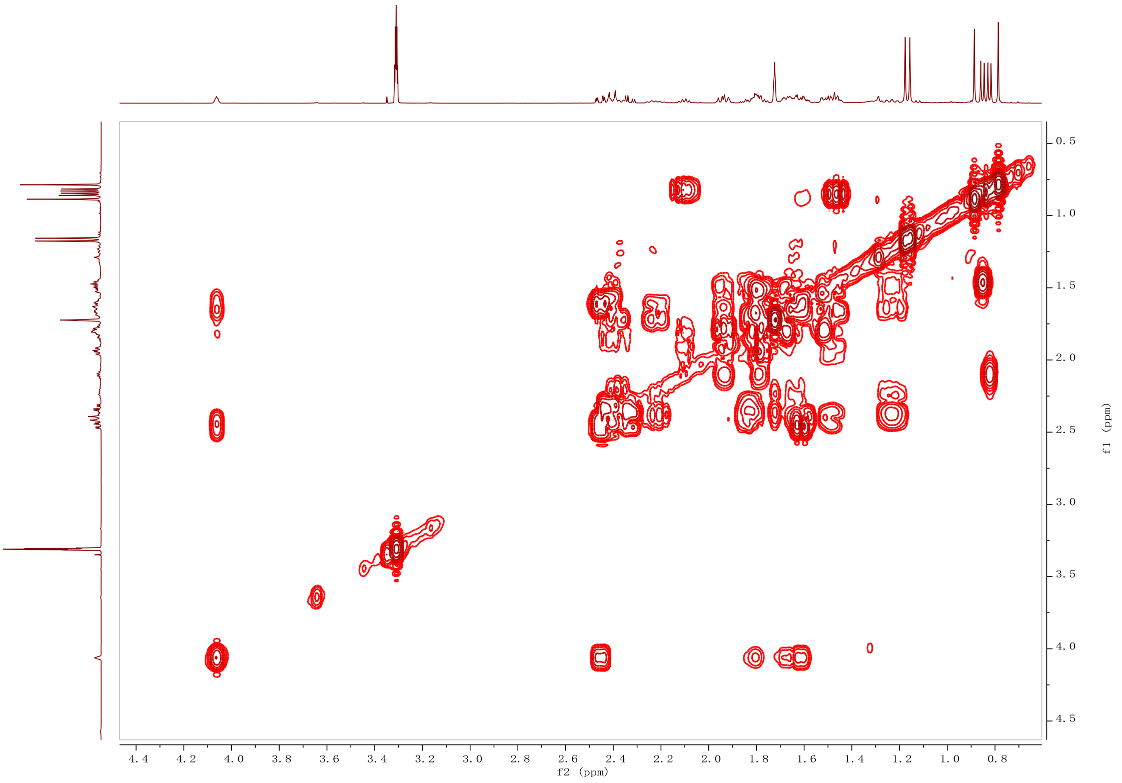

Fig.S18. ^{1}H,^{1}H COSY spectrum of compound **2** in CD3OD.

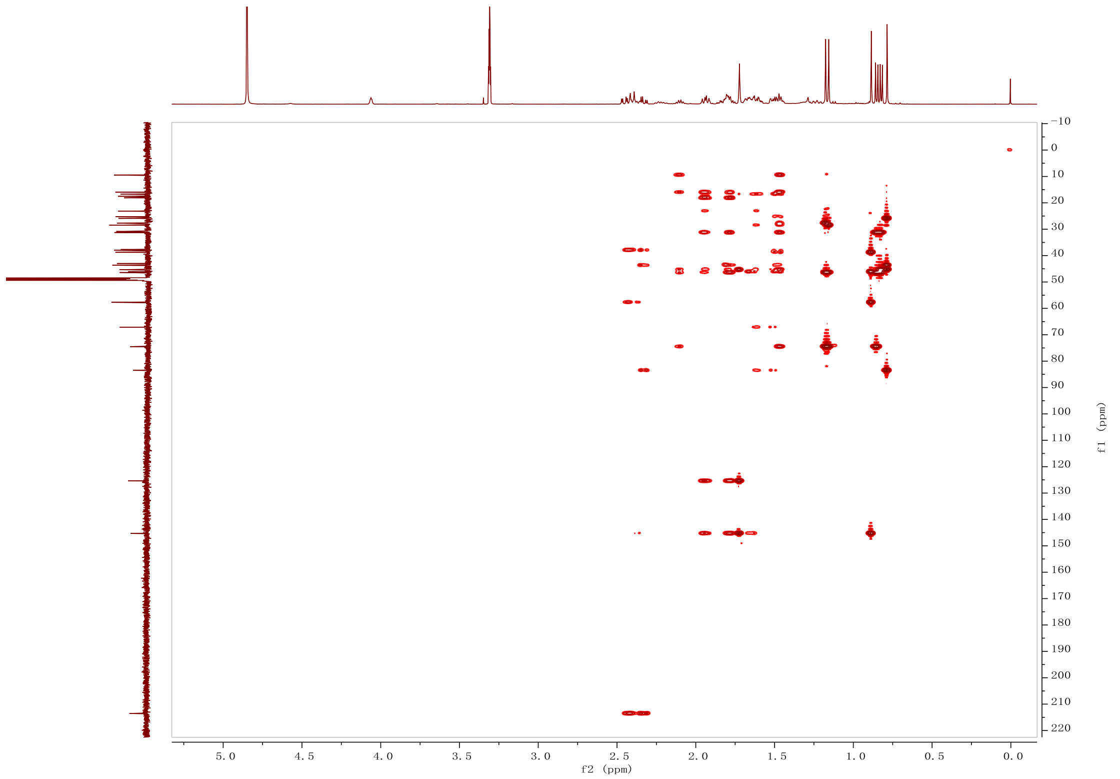

Fig.S19. HMBC spectrum of compound **2** in CD3OD.

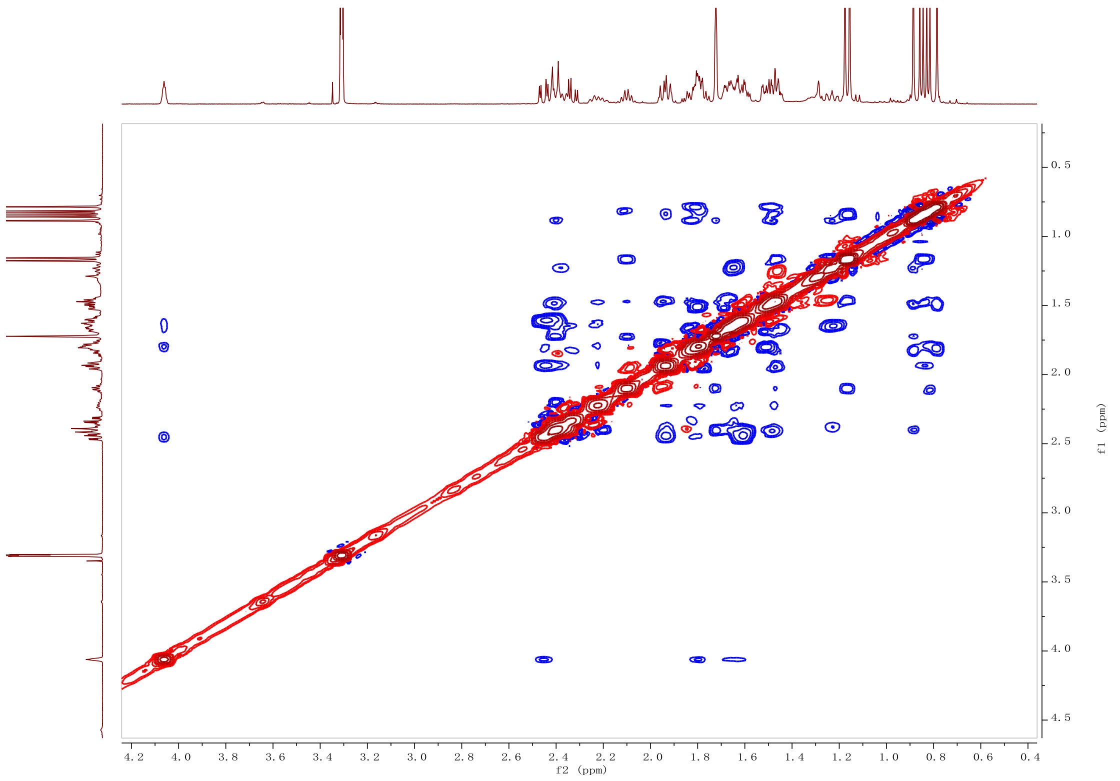

Fig.S20. NOESY spectrum of compound **2** in CD3OD.

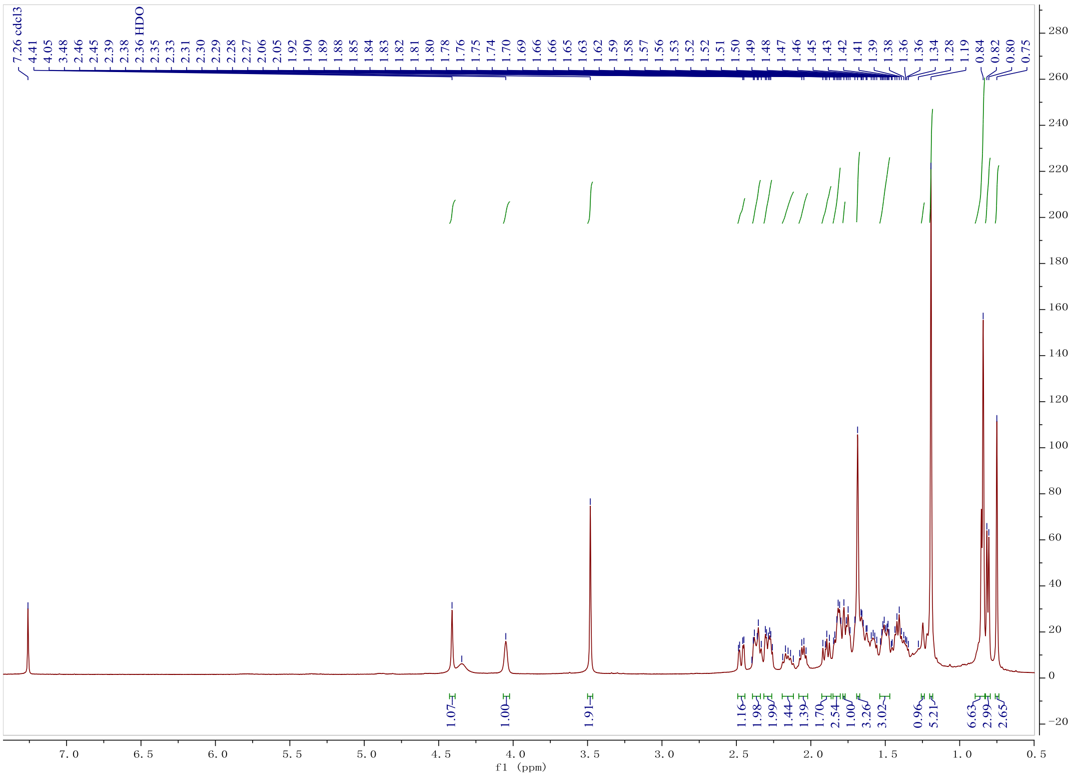

Fig.S21. ^{1}H NMR spectrum of compound **2** in $CDCl_3$.

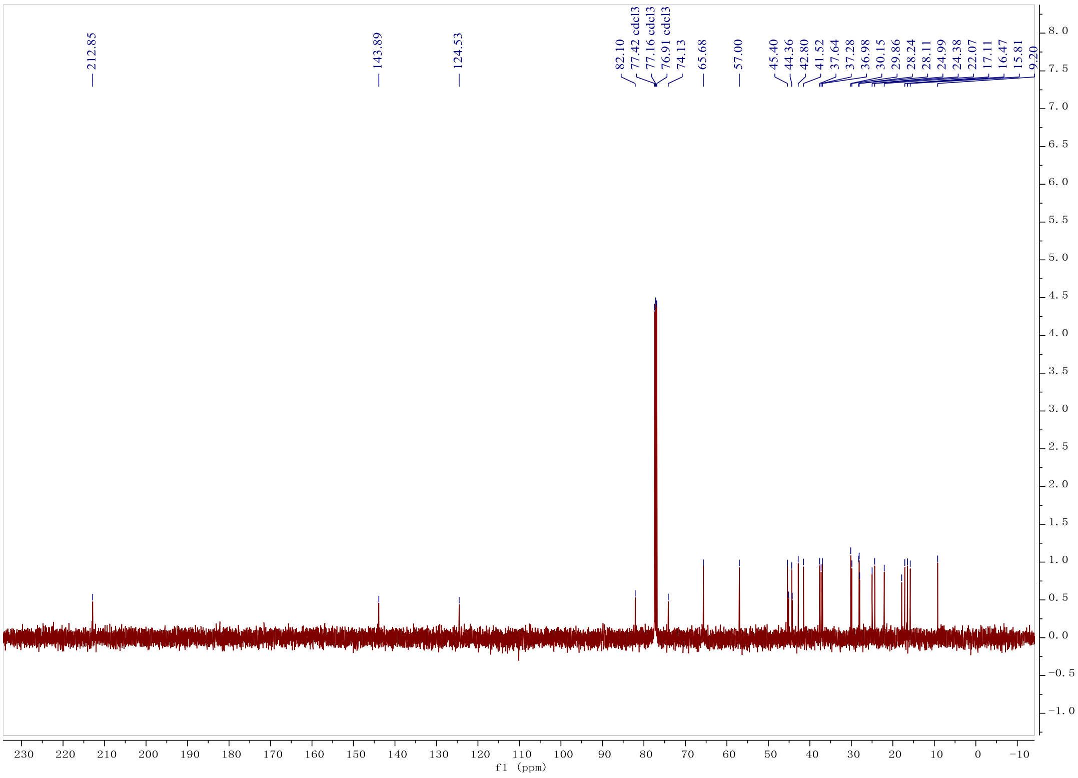

Fig.S22. ^{13}C NMR spectrum of compound **2** in $CDCl_3$

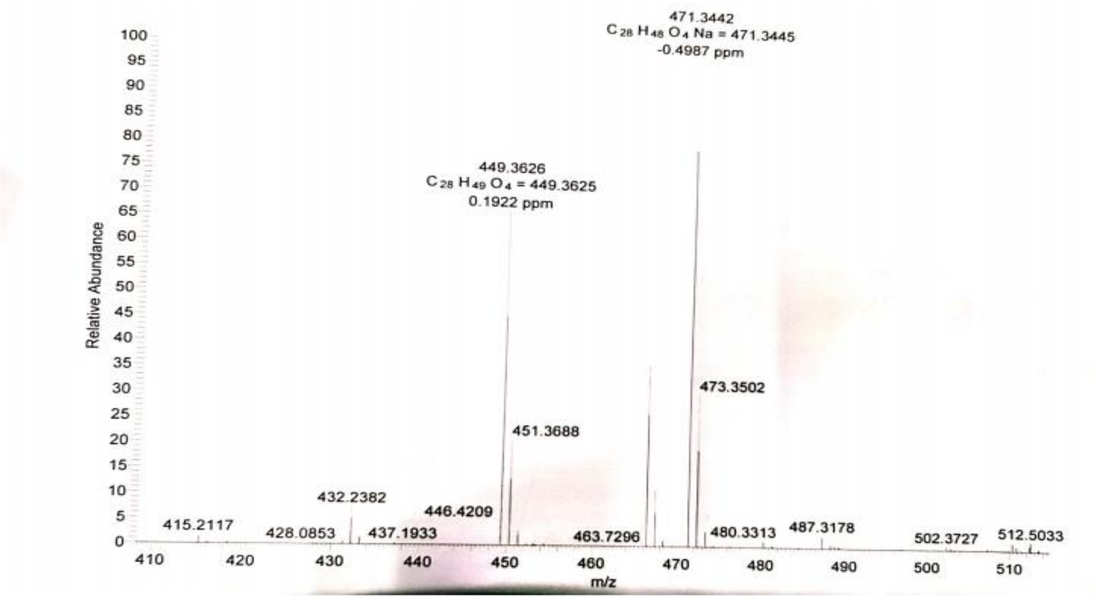

Fig.S23. HRESIMS spectrum of compound **3**.

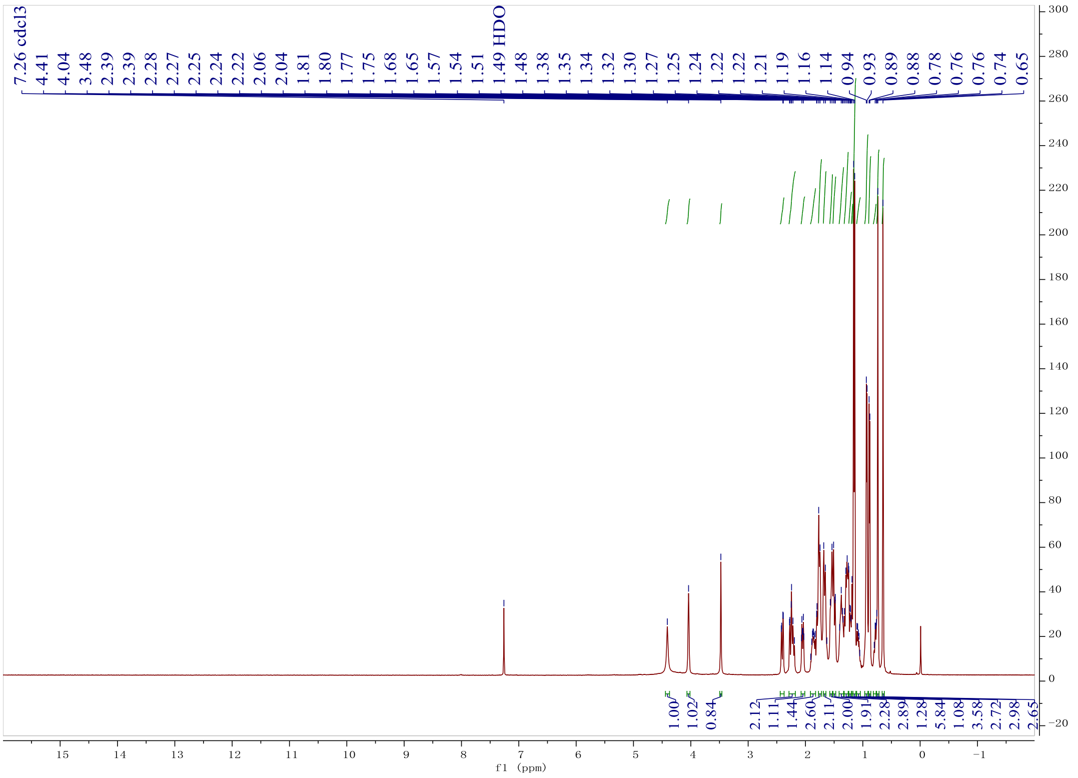

Fig.S24. ^{1}H NMR spectrum of compound **3** in $CDCl_3$.

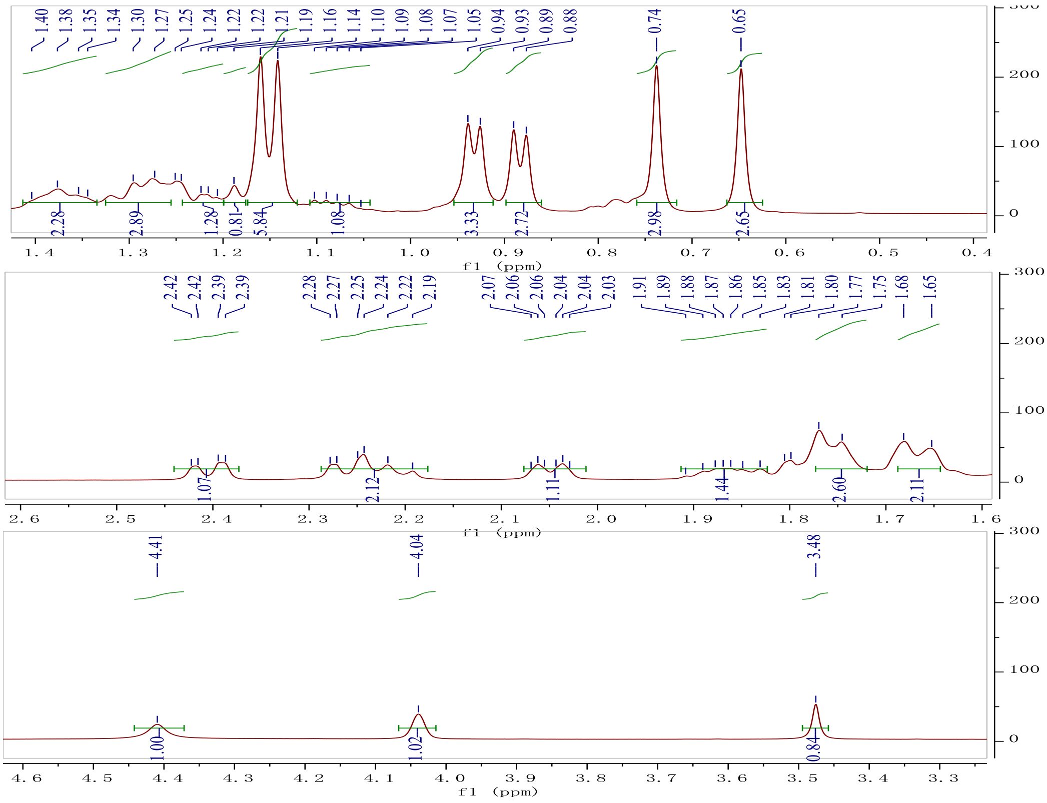

Fig.S25. The amplificatory ^{1}H NMR spectrum of compound **3** in $CDCl_3$.

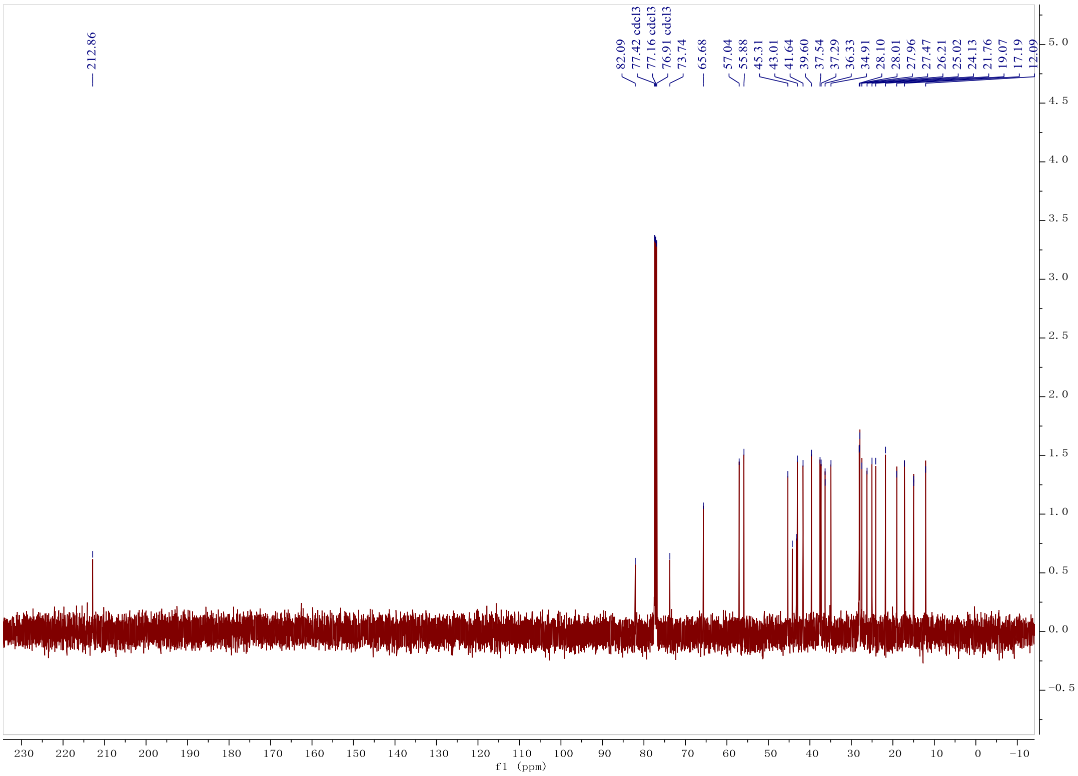

Fig.S26. ^{13}C NMR spectrum of compound **3** in $CDCl_3$.

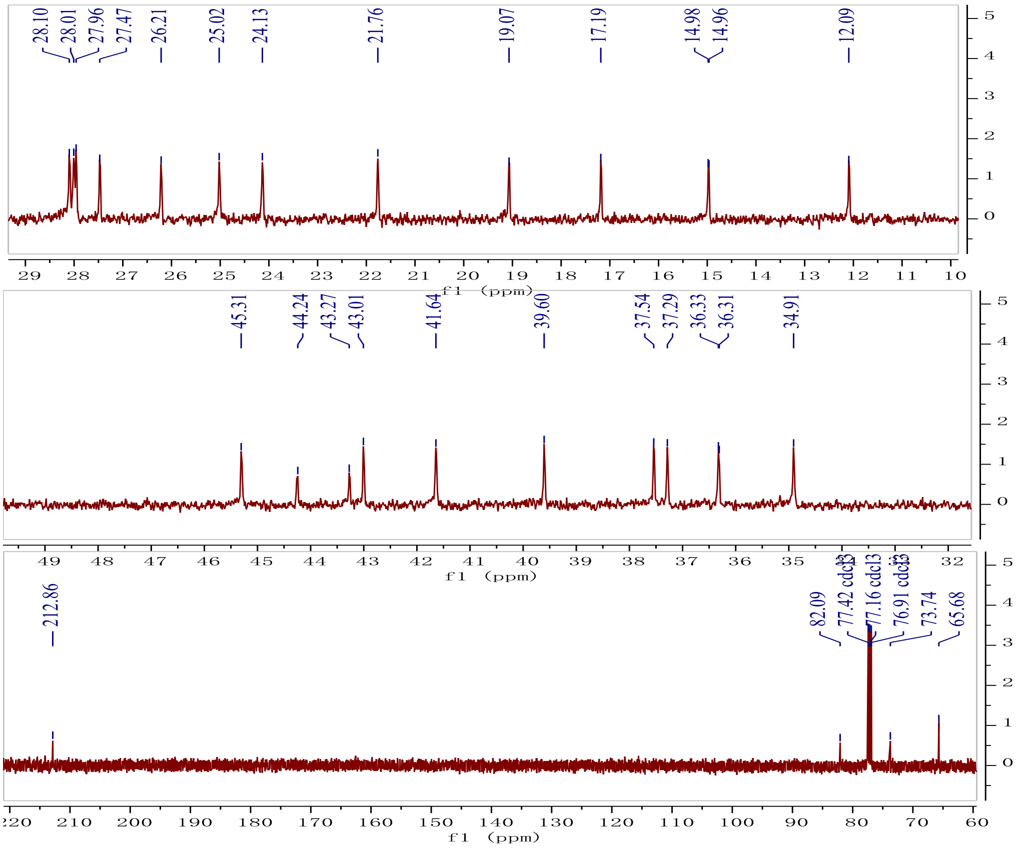

Fig.S27. The amplificatory ^{13}C NMR spectrum of compound **3** in $CDCl_3$.

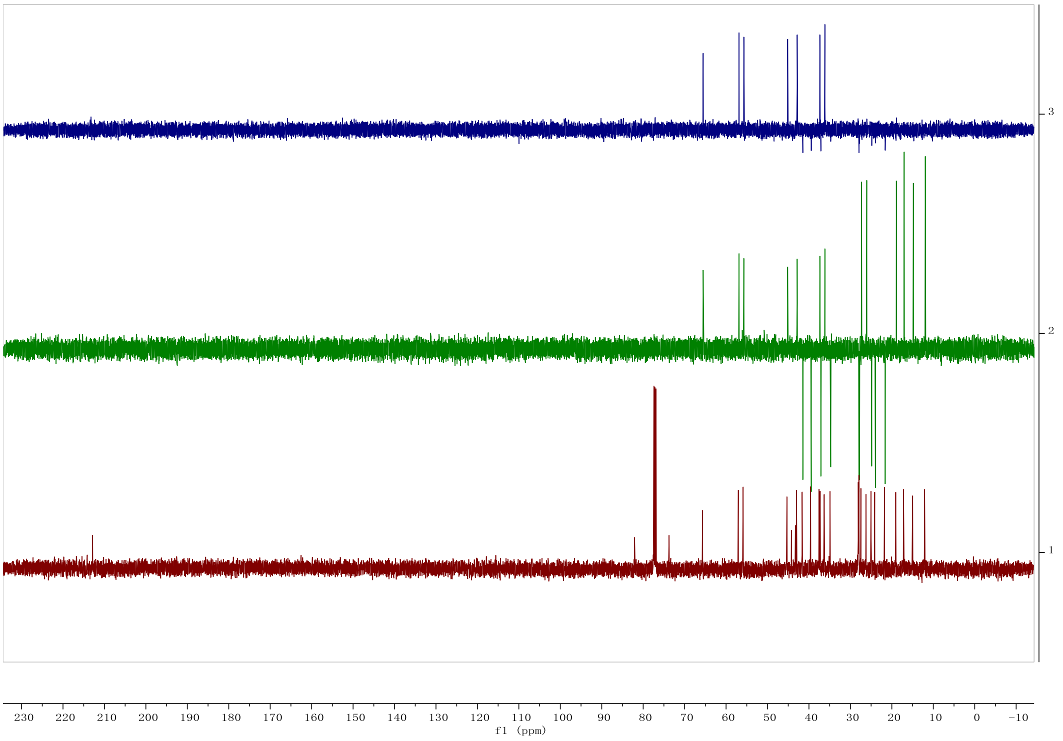

Fig.S28. DEPT spectrum of compound **3** in $CDCl_3$.

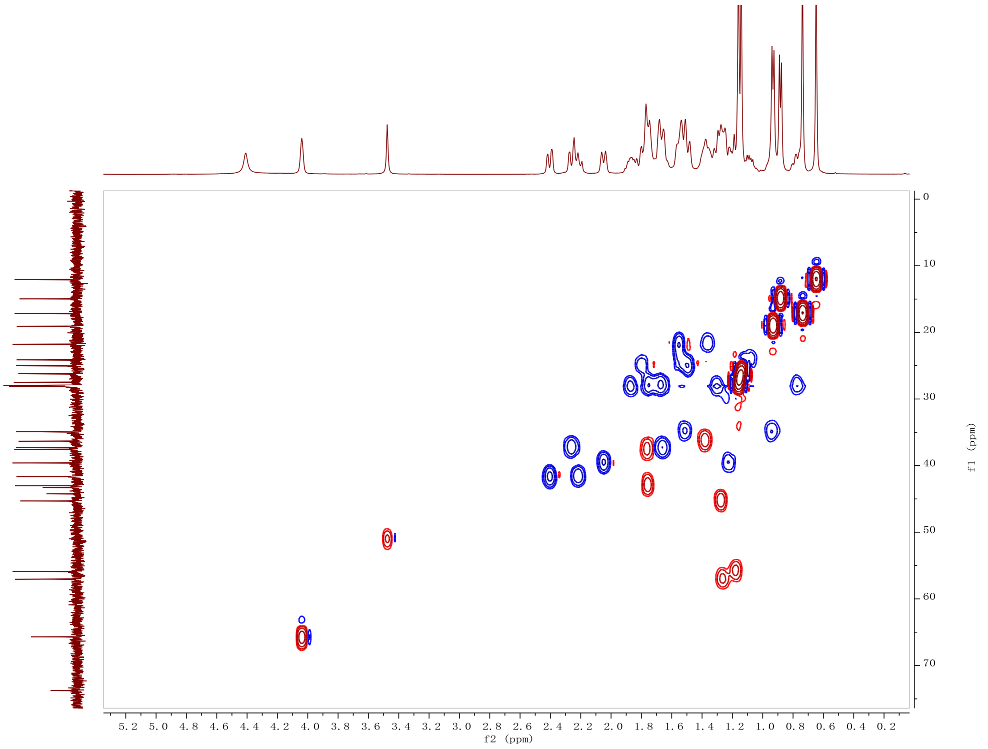

Fig.S29. HSQC spectrum of compound **3** in $CDCl_3$.

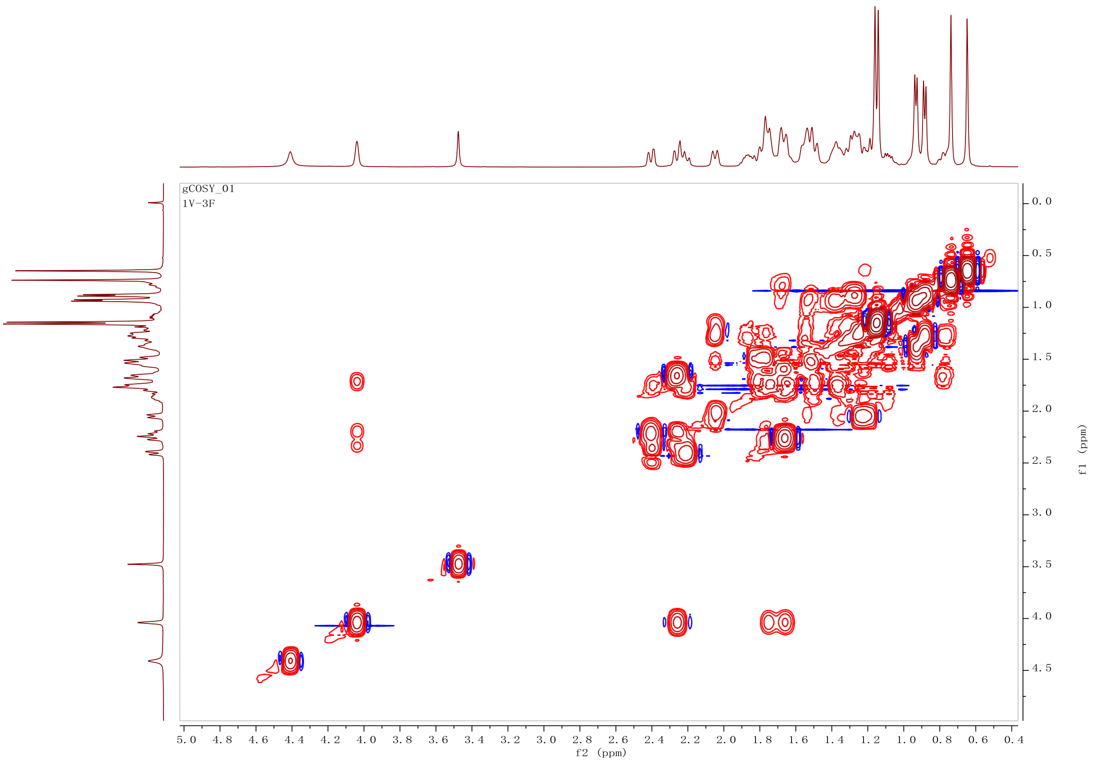

Fig.S30. $^{1}H,^{1}H$-COSY spectrum of compound **3** in $CDCl_3$.

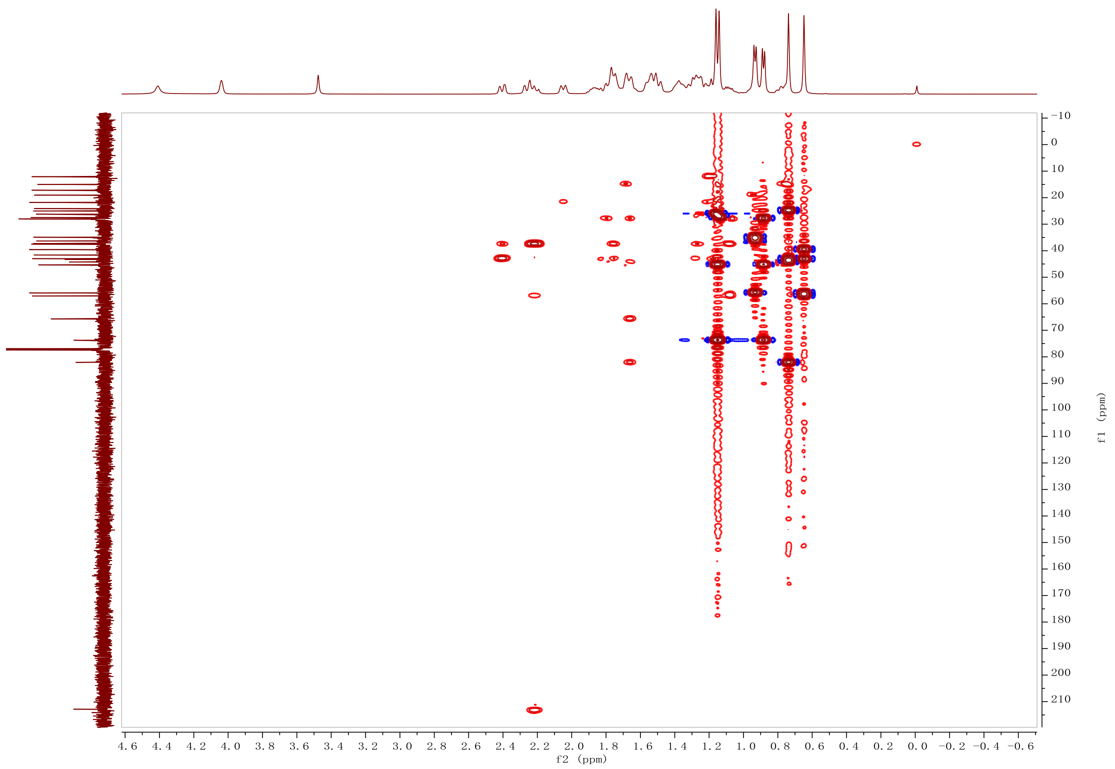

Fig.S31. HMBC spectrum of compound **3** in $CDCl_3$.

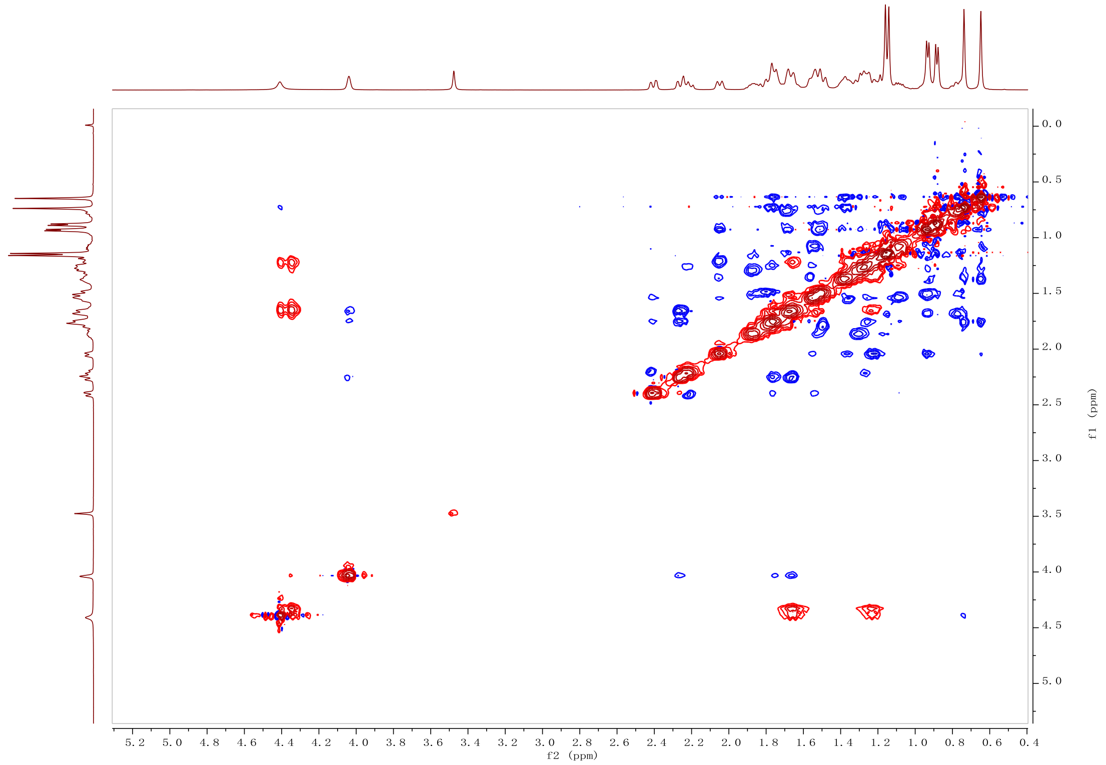

Fig.S32. NOESY spectrum of compound **3** in $CDCl_3$.

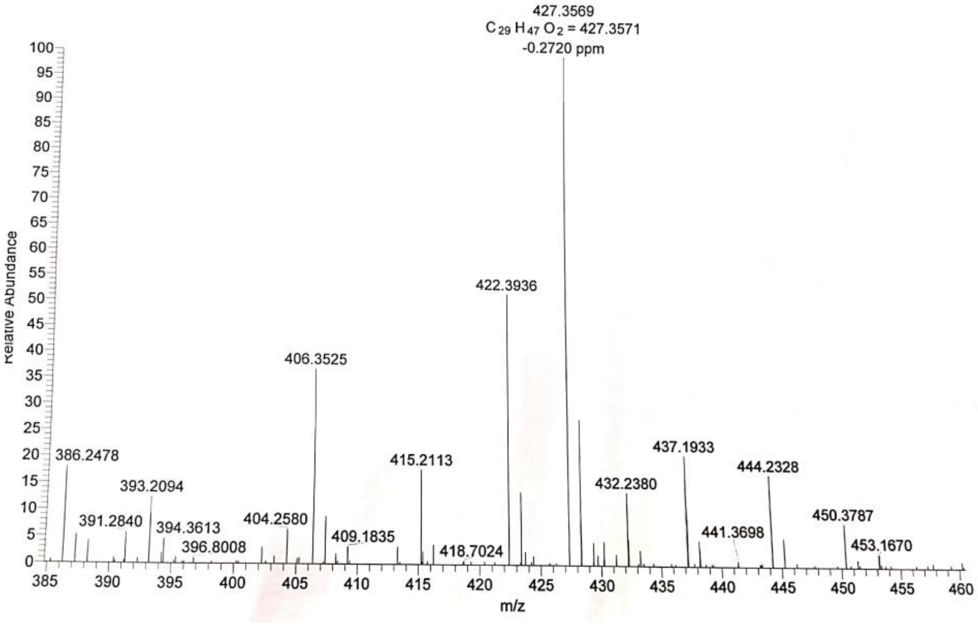

Fig.S33. HRESIMS spectrum of compound **4**.

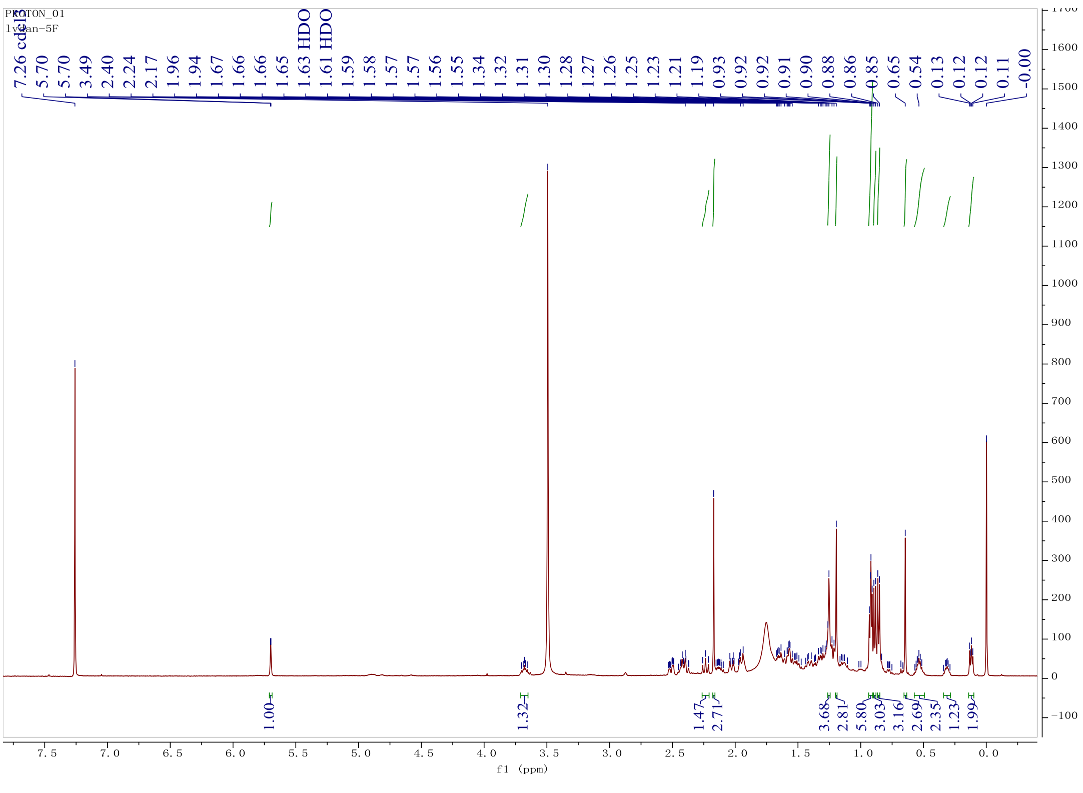

Fig.S34. ^{1}H NMR spectrum of compound **4** in $CDCl_3$.

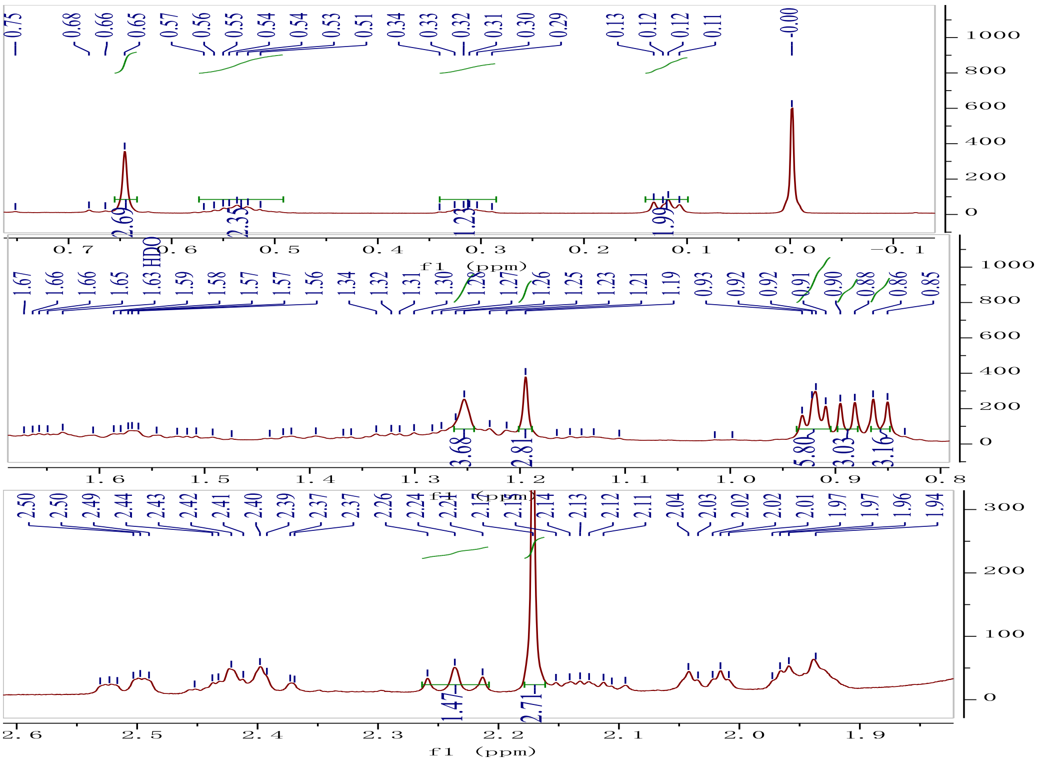

Fig.S35. The amplificatory ^{1}H NMR spectrum of compound **4** in $CDCl_3$

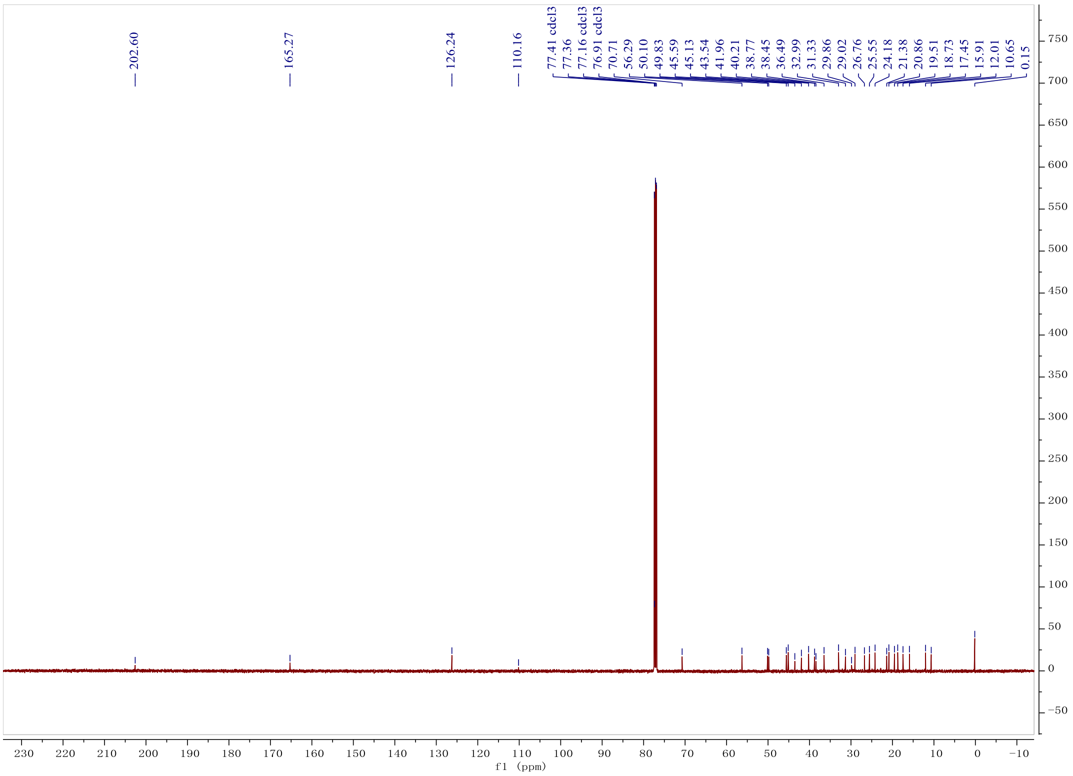

Fig.S36. ^{13}C NMR spectrum of compound **4** in $CDCl_3$.

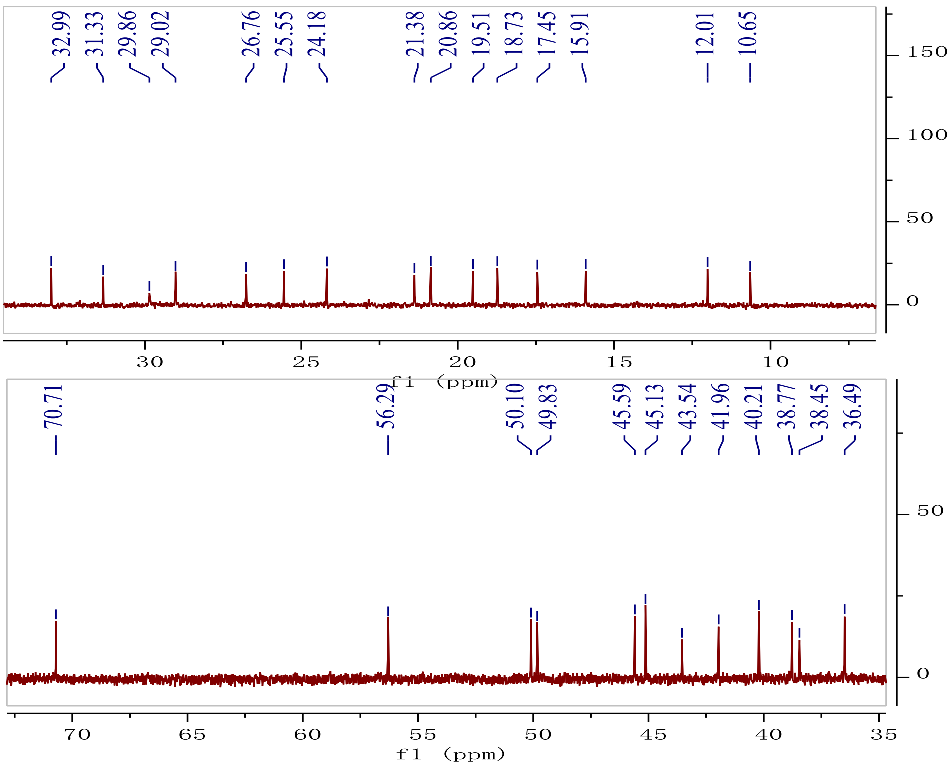

Fig.S37. The amplificatory ^{13}C NMR spectrum of compound **4** in $CDCl_3$.

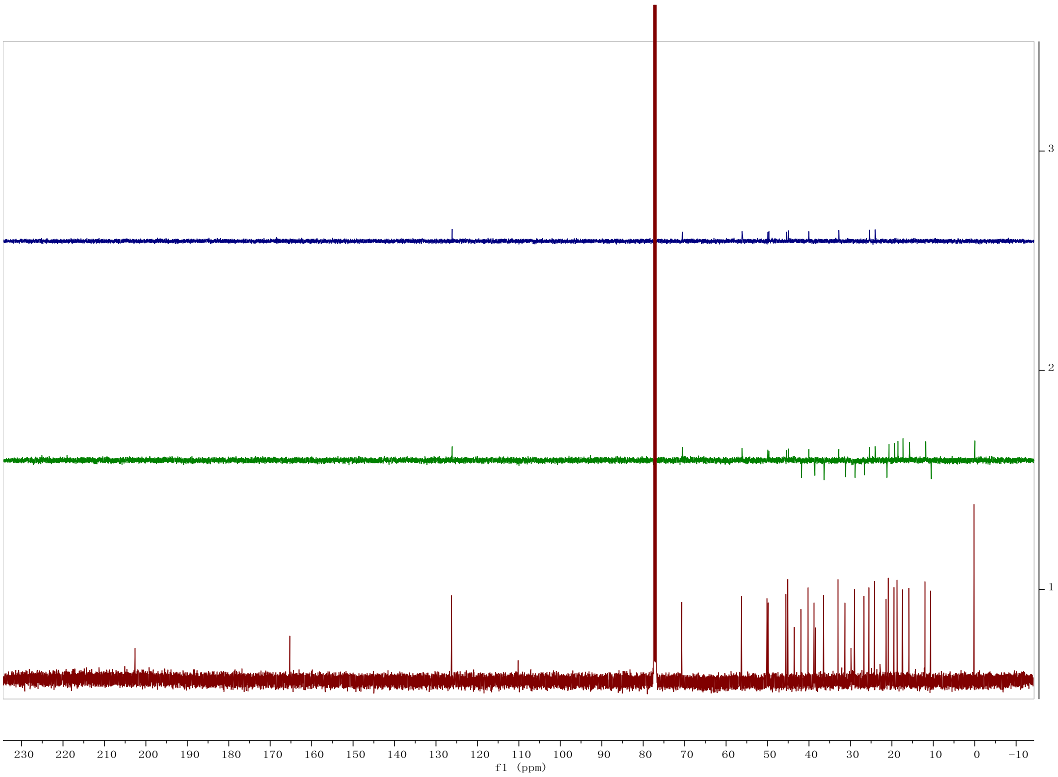

Fig.S38. DEPT spectrum of compound **4** in $CDCl_3$.

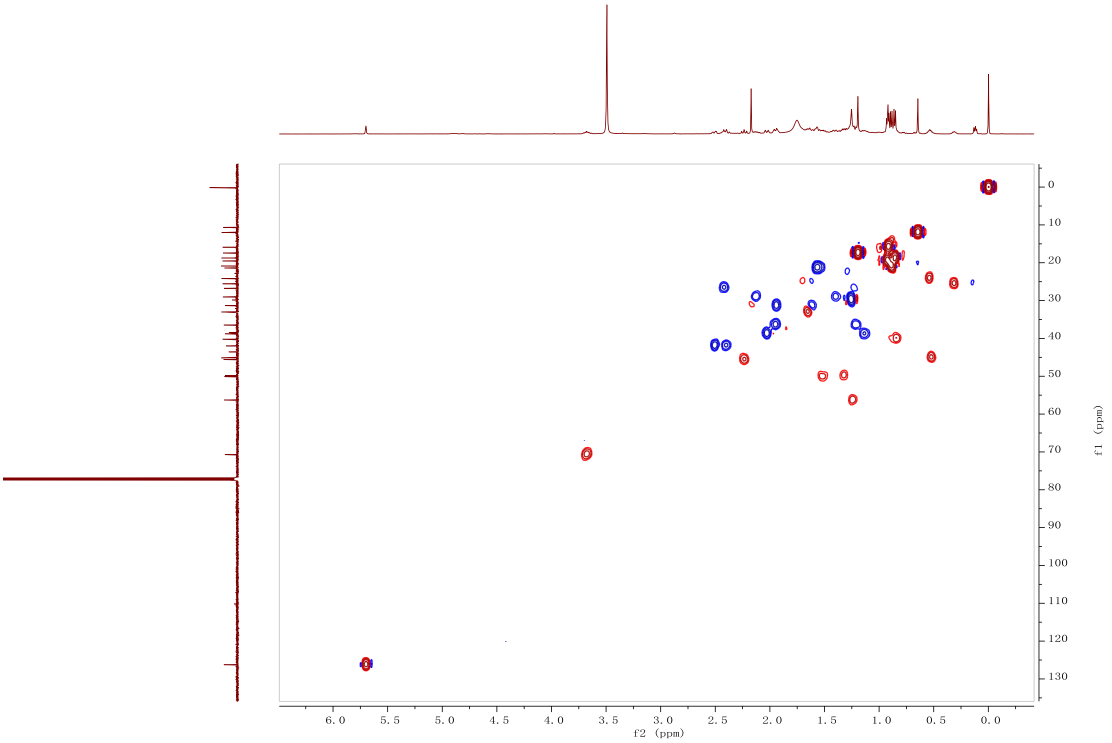

Fig.S39. HSQC spectrum of compound **4** in $CDCl_3$.

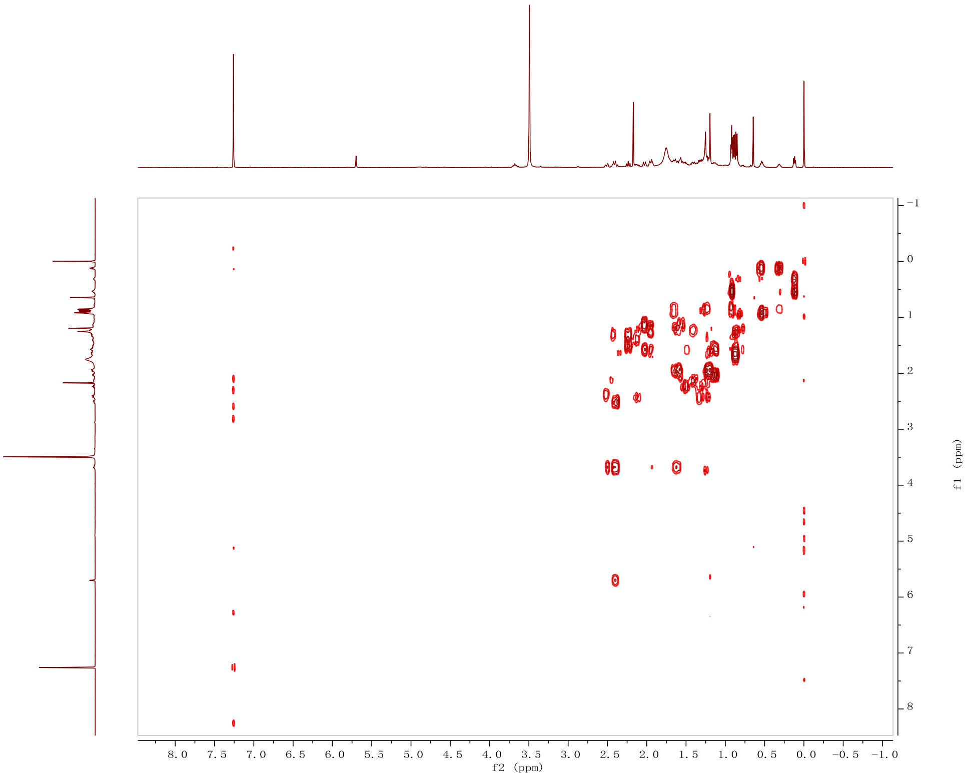

Fig. S40. $^1H,^1H$-COSY spectrum of compound **4** in $CDCl_3$.

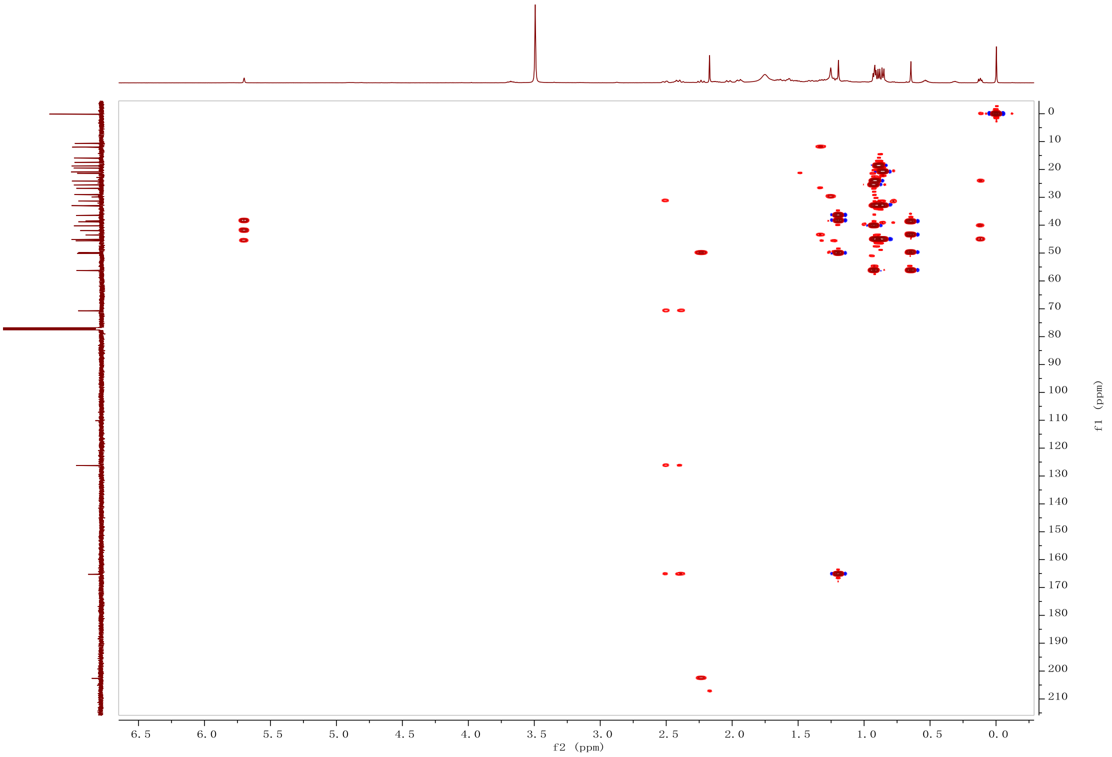

Fig. S41. HMBC spectrum of compound **4** in $CDCl_3$.

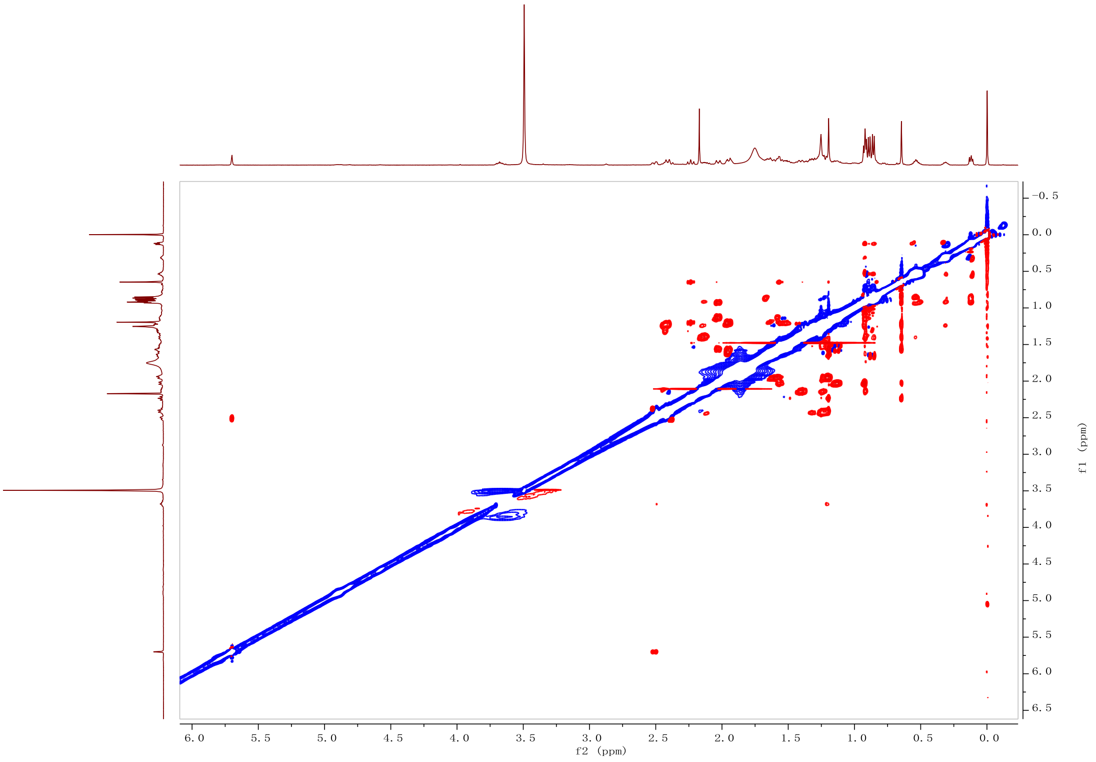

Fig. S42. NOESY spectrum of compound **4** in $CDCl_3$.

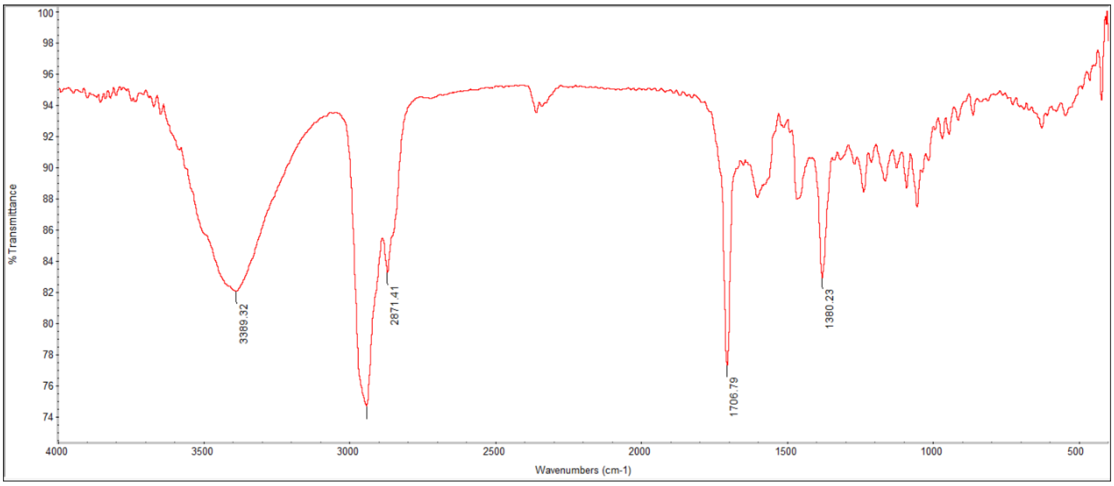

Fig. S43. IR (KBr disc) spectrum of compound **1**.

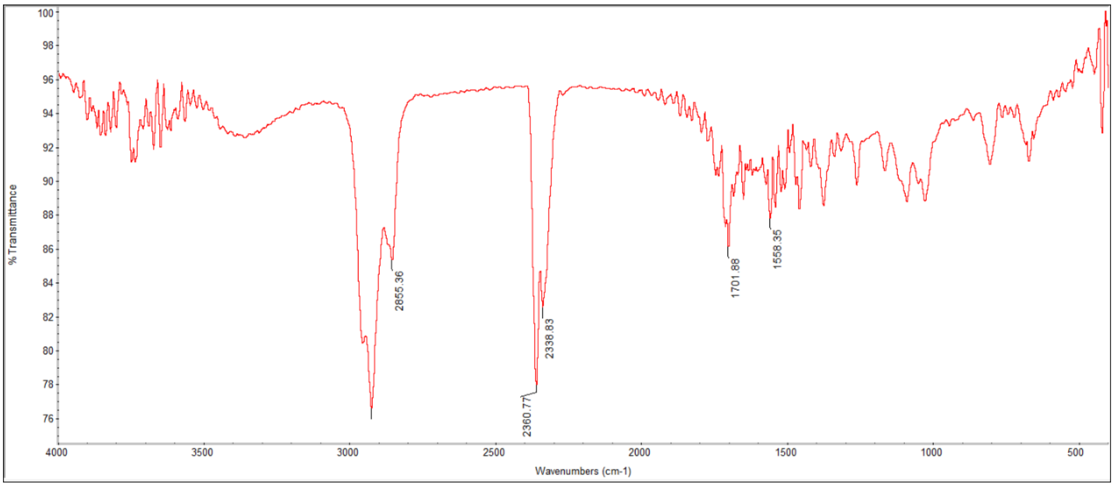

Fig. S44. IR (KBr disc) spectrum of compound **2**.

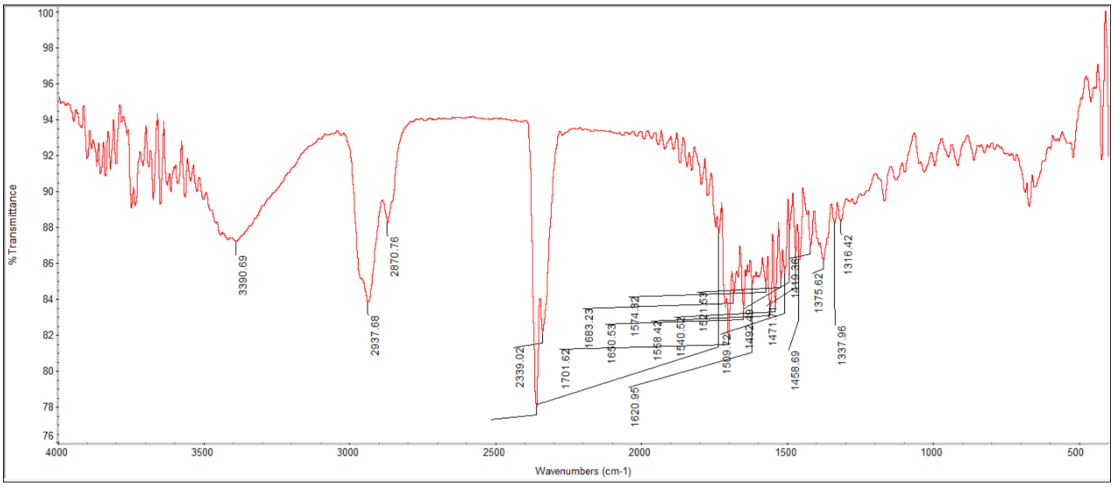

Fig. S45. IR (KBr disc) spectrum of compound **3**.

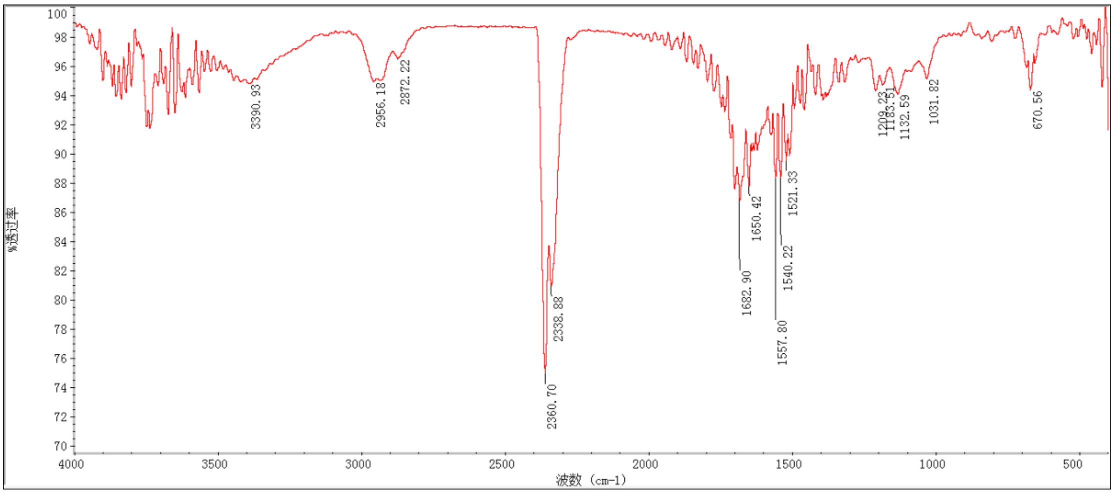

Fig. S46. IR (KBr disc) spectrum of compound **4**.
